# Propranolol and Capecitabine Synergy on Inducing Ferroptosis in Human Colorectal Cancer Cells: Potential Implications in Cancer Therapy

**DOI:** 10.3390/cancers17091470

**Published:** 2025-04-27

**Authors:** Shiekhah Mohammad Alzahrani, Huda Abdulaziz Al Doghaither, Hind Ali Alkhatabi, Mohammad Abdullah Basabrain, Peter Natesan Pushparaj

**Affiliations:** 1 Biochemistry Department, Faculty of Science, King Abdulaziz University, Jeddah 21589 P.O. Box 80200, Saudi Arabia; 2 Institute of Genomic Medicine Sciences, Faculty of Applied Medical Sciences, King Abdulaziz University, Jeddah P.O. Box 21589, Saudi Arabia; 3 Department of Biological Science, College of Science, University of Jeddah, Jeddah P.O. Box 21589, Saudi Arabia; 4 Department of Medical Laboratory Technology, Faculty of Applied Medical Sciences, King Abdulaziz University, Jeddah P.O. Box 21589, Saudi Arabia

**Keywords:** colorectal cancer, propranolol, capecitabine, transcriptomic, metabolomic, ferroptosis, migration

## Abstract

Background/Objectives: Colorectal cancer (CRC) is a significant global health issue with rising incidence and mortality rates. In oncology, drug repurposing has emerged as a promising therapeutic strategy in conjunction with conventional treatments. This study aimed to evaluate the potential of repurposing propranolol (PRO), a beta blocker, for the treatment of CRC cell lines (HCT-116 and HT-29), both as a monotherapy and in combination with capecitabine (CAP). Methods: Effects of mono- and combination therapies on viability, combination index, morphology, and cell death induction of CRC cells were assessed. Transcriptome analysis of HT-29 cells was performed using RNA sequencing. Metabolite profiling was conducted, and changes in biochemical parameters were evaluated using flow cytometry and biochemical analyses. Results: The combination index showed that HT-29 cells were the most responsive to the combined treatment, even with *PIK3CA*, *B-RAF* (V600E), and *TP53* mutations. Moreover, ferroptosis was synergistically activated in the combined group of HT-29 in comparison to control. Furthermore, we observed an increase in OXPHOS metabolites, along with elevated intracellular and mitochondrial ROS, disruption of mitochondrial membrane potential, and greater levels of malondialdehyde (MDA) in the HT-29 combined group, which are the features of ferroptosis. Furthermore, ferroptosis induction was coupled with necroptosis, as indicated by RNA-sequencing data. Combination therapy inhibited cell migration and enhanced the immune response of HT-29 cells. Conclusions: These findings suggest that PRO is promising as a potential adjuvant therapy in combination with CAP for the treatment of CRC. Only HT-29 cells with the *B-RAF* (V600E) mutation showed promising findings in this study.

## Simple Summary

Colorectal cancer (CRC) is a lethal tumor worldwide. In oncology, drug repurposing has emerged as a promising therapeutic strategy in conjunction with classical treatments. Propranolol has been proposed as an anti-cancer therapeutic agent for several tumors. We performed transcriptomic and metabolomic studies, as well as several biochemical parameter assays, to evaluate the antitumor effects of propranolol and/or capecitabine treatments on CRC cell lines. Our study identified novel metabolites and transcripts as potential therapeutic targets for propranolol and capecitabine treatment. It reflects the importance of precision medicine and suggests that propranolol holds promise as a potential adjuvant therapy in combination with capecitabine for the treatment of CRC.

## 1. Introduction

Colorectal cancer (CRC) is a global health burden, ranking second in mortality and third in incidence among malignant tumors worldwide [1]. This disease is characterized by its heterogeneous nature, driven by complex molecular genetics and epigenetic mechanisms [2]. In different types of tumors, including CRC, driver mutations in proto-oncogenes and tumor suppressor genes influence gene expression profiles, epigenetic shapes, and metabolic profiles [3]. Certainly, this metabolic reprogramming is recognized as a critical hallmark of most cancers, which is required for tumor adaptation and for sustaining their urgent malignant growth, metastasis, and evasion of apoptosis [3,4,5,6]. The majority of CRC tumors are driven by the activation of oncogenic *KRAS*, *BRAF*, and *PIK3CA* mutations and in-activation mutations of the tumor suppressor *TP53*, which have been strongly linked to metabolic reprogramming in CRC [3,4,5] and drug resistance [6]. In addition, crosstalk between oncogenic signaling pathways is correlated with CRC development [3].

In the realm of CRC treatment, capecitabine (CAP) is a 5-floururacil-based chemotherapeutic agent which belongs to the antimetabolites class. CAP is a tumor-selective cytotoxic agent that is selectively activated by the thymidine phosphorylase (TP) enzyme. This antimetabolite was designed as a prodrug to form 5-FU preferentially in situ or at the tumor site and then inhibit DNA synthesis. CAP is a cornerstone in both primary and metastatic CRC management because of its efficacy and tolerability profiles [7,8,9]. However, drug resistance to 5-FU-based chemotherapy is one of the greatest challenges in the management of CRC. It can be acquired or intrinsic during treatment and is considered to occur in ~50% of patients with metastatic CRC. Chemoresistance to CAP may be due to various factors that are relevant to the molecular features (genetic and/or epigenetic) and metabolic characteristics of patients with CRC [9,10]. To counter chemoresistance, new treatment strategies are being employed in cancer therapy, such as the development of novel adjuvant therapies and drug combinations [11]. Specific drug combinations in cancer treatment may provide additional positive effects, such as enhancement of tumor therapy efficiency, which may be absent in a single drug [12].

Drug repurposing is another new therapeutic approach in the field of oncology [11]. This approach is an alternative therapy used in the clinical field that involves reusing existing FDA-approved drugs for therapeutic purposes other than their main ones based on their identified roles. In the clinical setting, this strategy offers many advantages over the development of new agents with a well-known safety profile, accessibility, and low cost [11,12,13,14].

Propranolol (PRO) is one of the most promising drugs for oncology, including CRC [11,15,16,17,18,19]. PRO is defined as a non-cardio-selective β-adrenergic receptor (β-AR) antagonist that is used mainly for the treatment of cardiovascular diseases [17,20]. Moreover, PRO is a beta-blocker (βB) agent that acts as the blocking agent of the beta-adrenergic signaling pathway [17,21]. Coelho et al. reported that PRO is the most potent βB agent for inhibiting adrenaline-induced proliferation of CRC cells [17]. PRO is well-known for its high safety profile and tolerance in clinical practice [22,23]. Hence, PRO has been investigated in preclinical and clinical studies as a repurposed drug for cancer treatment, including CRC [15,16,17,18,19]. PRO exerts antitumor effects by inhibiting angiogenesis and inducing apoptosis [24]. Many clinical trials have suggested that PRO could also be implemented as a cardioprotective agent against the potential cardiac complications of chemotherapy and radiotherapy, especially in elderly patients with cancer [25], and may be used as a promising novel adjuvant/coadjuvant (in-combination) therapy for CRC [23,25,26]. However, some clinical trials of single targeted agents have faced challenges resulting from toxicity and/or the development of resistance. One strategy to address these challenges involves the synergistic combination of targeted agents [27].

Here, we aimed to utilize the HCT-116 human colon carcinoma cell line (mut *PIK3CA*, mut *KRAS^G13D^*, and MMR deficient) and HT-29 human colorectal adenocarcinoma cell line (mut *PIK3CA*, mut *BRAF^V600E^*, and mut *TP53*) as CRC preclinical models to investigate the therapeutic potential of PRO in combination with CAP. Notably, to the best of our knowledge, no previous in vivo or in vitro studies have explored the anti-cancer effects of PRO and CAP in a dual therapy approach for CRC. Therefore, the current study was designed to investigate the synergistic effects of PRO and CAP drugs on CRC to identify a potential future treatment approach. Furthermore, we extended our investigation by employing an omics approach to assess the impact of these therapeutic agents on the transcriptomic and metabolomic profiles in CRC.

## 2. Materials and Methods

### 2.1. Cell Culture

The HCT-116 human colon carcinoma cell line and the HT-29 human colorectal adenocarcinoma cell line were obtained from King Faisal Specialist Hospital and Research Center in Jeddah (KFSHRC-J). CRC cell lines were cultured in high-glucose DMEM supplemented with 10% fetal bovine serum (FBS) and 1% penicillin/streptomycin. The cell lines were routinely maintained in culture at 37 °C in a humidified atmosphere containing 5% CO_2_.

### 2.2. Cell Viability Analysis

The 3-(4,5-Dimethylthiazol-2-yl)-2,5-diphenyltetrazolium bromide (MTT) assay (Invitrogen, Thermo Fisher Scientific, USA) was used to assess the cytotoxicity of individual drugs or drug combinations in the cell lines. Briefly, cells were seeded in a 96-well plate at a density of 10 × 10^3^ cells/well in triplicate. The following day, the growth medium was replaced with increasing doses of the indicated drugs (PRO and CAP monotherapies) for 48 h. MTT assay was performed as previously described [28]. The absorbance of the purple color was measured at 570 nm using a SpectraMax i3 microplate reader (Molecular Devices, LLC, San Jose, CA, USA) at 570 nm.

### 2.3. Drug Combination Studies

The Chou–Talalay method of dual-drug combination was used to measure synergistic therapeutic effects, minimizing the dose and toxicities. The IC_50_ values from the dose–response curves that were calculated using GraphPad Prism version 9.0 were used for the combined therapy. The combination index value (CI) was calculated using CompoSyn 1.0 software (www.combosyn.com, accessed on 23 July 2022), which is considered the standard measure of combination effect based on the Chou–Talalay method [29]. Cells were seeded at 10 × 10^3^ cells/well in 96-well plates. On the next day, the cells were treated with PRO alone, CAP alone, or PRO with CAP at five concentrations (0.25 × IC_50_, 0.5 × IC_50_, 1 × IC_50_, 2 × IC_50_, and 4 × IC_50_) for 48 h. The cytotoxicity of the drug combination was determined using the MTT test. The CI experiments were performed in triplicate (n = 3).

### 2.4. Morphological Images

Cells were seeded in 6-well plates at 2 × 10^5^ cells/well (HCT-116) and 3 × 10^5^ cells/well (HT-29) and incubated for 24 h at 37 °C. After treatment with mono- or combination therapies based on IC_50_ values for 48 h, cell morphology was assessed using a Nikon Eclipse inverted microscope (Nikon Eclipse, Tokyo, Japan) at 10× magnification. The appearance of the treated cells was compared with that of the control cells.

### 2.5. The Detection of Cell Death

Annexin V-FITC/PI (ab14085, Abcam, Cambridge, UK) was used to detect cell death using a FACSCanto^TM^ II flow cytometer (BD Biosciences, Franklin Lakes, NJ, USA) [30]. The cells were plated and exposed to mono- and dual therapies for 48 h, as described in the Section 2.4 Morphological Images. Cells were stained with Annexin V and analyzed by flow cytometry according to the manufacturer’s instructions. A minimum of 10,000 events were acquired for the analysis.

### 2.6. Transcriptomic Study

#### 2.6.1. RNA Extraction

Briefly, the HT-29 cells were seeded at 8 × 10^5^ cells in 25 cm^2^ flasks. After treatment with PRO and/or CAP, cells were collected, and total RNA was extracted using an RNA isolation kit (Haven Scientific, Jeddah, Saudi Arabia) following the manufacturer’s instructions. The purity of the extracted RNA was measured using NanoDrop *DeNovix* DS-11 (Thermo Fisher Scientific, Waltham, MA, USA). The RNA-containing eluate was stored at −80 °C for RNA sequencing analysis. The RNA samples preserved in ambient tubes were sent to Novogene for RNA sequencing [31].

#### 2.6.2. RNA Library Construction

Quality Control and Sequencing. Messenger RNA was purified from the total RNA using poly T oligo-attached magnetic beads. After fragmentation, the first-strand complementary DNA (cDNA) was constructed using random hexamer primers. Subsequently, second-strand cDNA was synthesized using dTTP for a nondirectional library. Following end repair, A-tailing, adapter ligation, size selection, amplification, purification, nondirectional library, and RNA sequencing were performed.

To check the library, Qubit and real-time polymerase chain reaction (PCR) were used for quantification, and a bioanalyzer was used for size distribution detection. Quantified libraries were pooled and sequenced on an Illumina platform based on the effective library concentration and amount of data. Clustering of the index-coded samples was performed according to the manufacturer’s instructions. After cluster generation, the prepared libraries were sequenced on an Illumina platform, and paired-end reads were generated. Raw data (raw reads) in FASTQ format were processed using fastp software. In this step, clean data (clean reads) were acquired by excluding reads containing adapters, poly-N, and low-quality reads from the raw data. Therefore, downstream analyses were based on high-quality, clean data. The index of the reference genome was created using Hisat2 v2.0.5, and the clean paired-end reads were aligned to the reference genome using Hisat2 v2.0.5. To count the read numbers mapped to each gene, feature Counts v1.5.0-p3 was used. Fragments per kilobase of transcript per million mapped fragments (FPKM) for each gene were calculated according to the length of the gene and read counts mapped to this gene.

#### 2.6.3. Identification of Differentially Expressed Genes (DEGs)

The DESeq2 R package (1.20.0) was used for differential expression analysis. It provides a statistical analysis for determining differential expression in digital gene expression data using a model that depends on a negative binomial distribution. Genes with a *p*-value ≤ 0.05 were considered as DEGs.

#### 2.6.4. Enrichment Analysis

The Kyoto Encyclopedia of Genes and Genomes (KEGG) and Reactome were used to identify the pathways enriched by DEGs. KEGG pathway enrichment analysis is a database (http://www.genome.jp/kegg/, accessed on 23 July 2022) used for understanding high-level functions of the biological system from molecular-level information. The Reactome database provides information on many reactions and biological pathways in the human model species. The above analysis (including sample and data analyses) was performed using Novogene [31].

### 2.7. Global Untargeted Metabolomics Profiling Study Using LC-MS/MS

#### 2.7.1. Metabolites Extraction

A total of 8 × 10^5^ cells/T25-flask were seeded into the HT-29 cell line. After treatment, the cells were collected. Metabolite extraction from treated and untreated cells was immediately performed by lysing the cells using a tissue homogenizer with an ice-cold solvent comprising water: acetonitrile: methanol (H_2_O: ACN: Meth) at a ratio of 1:2:2 *v*/*v*, vigorously vortexed, and then incubated at −20 °C for 24 h. The lysate was centrifuged at 13,000 rpm for 15 min at 4 °C [32]. LC-MS/MS was used to separate the total cellular metabolites from treated and untreated HT-29 cells. Three replicate samples were subjected to metabolomic analysis (n = 3).

#### 2.7.2. HPLC Workflow

Ten microliters of each metabolite extract were injected into an HPLC column (Hypersail gold column C18 Part No: 25005-104630) at a flow rate of 0.2 mL/min. The mobile phase consisted of 99.9% methanol in formic acid and 0.1% formic acid (0.1% *v*/*v*). The gradient elution program was set to range from 5% to 30% for 30 min, 30% to 50% for 10 min, 50% for 10 min, and finally 50–95% for 20 min. Finally, separation was performed with an overall runtime of 70 min at a column temperature of 30 °C [32].

#### 2.7.3. HPLC-Mass Spectrometry

The injected samples were analyzed using an LTQ XL™ linear ion trap LC-MS/MS instrument (Thermo Fisher Scientific, Waltham, MA, USA) with the following MSn parameter settings: full scanning mode varied from 80 to 1000 *m*/*z*. In this case, helium was utilized as the buffering gas and nitrogen was used as the sheath gas, with a flow rate of 40 arbitrary units. The capillary temperature was set at 270 °C with a voltage of 4.0 V, and the spray voltage was set at −3.0 kV [32,33,34].

#### 2.7.4. Data Processing and Analysis

Open access to the XCMS online database (https://xcmsonline.scripps.edu, accessed on 23 July 2022) was used to analyze the raw files of HPLC-MS data and to detect chromatographic peaks. Then, the processed peak list was exported as a CSV file format or compatible format for further analysis. The resultant peaks were identified against human metabolites in the Human Metabolome Database (HMDB) (www.hmdb.ca, accessed on 23 July 2022). Next, the feature table was annotated with metabolite names. This feature table was used to conduct data statistics and pathway analyses using MetaboAnalyst 5.0 (www.metaboanalyst.ca, accessed on 23 July 2022) [32,33,34,35].

### 2.8. Biochemical Validation Assays

#### 2.8.1. Measurement of Intracellular Reactive Oxygen Species

ROS levels were measured using an H2DCFDA kit (ab113851, Abcam, Cambridge, UK) [36,37] on a BD FACSAria^TM^ III flow cytometer (BD Biosciences, Franklin Lakes, NJ, USA). Briefly, cells were seeded in 12-well plates at a density of 1 × 10^5^ cells/well for HT-29. The collected cells were stained and analyzed according to the manufacturer’s instructions. Subsequently, the fluorescence intensity of DCF was detected by flow cytometry at excitation and emission wavelengths of 485 and 535 nm, respectively. In addition, a proper fluorescent imaging protocol was maintained, as described in the H2DCFDA kit. Fluorescence images were captured using an *EVOS FL* fluorescence microscope (Life Technologies, Carlsbad, CA, USA).

#### 2.8.2. Measurement of Mitochondrial Reactive Oxygen Species (Mito ROS)

This assay was performed to further examine whether cell death was mediated by mitochondrial oxidative stress via excess Mito ROS formation [38]. HT-29 cells (1 × 10^4^ cells/well) were seeded into 96-well plates and incubated overnight at 37 °C in 5% CO_2_. Following treatment with PRO and/or CAP, the MitoROS assay was performed according to the manufacturer’s instructions using the MitoROS 580 kit (ab219943, Abcam, Cambridge, UK). Red fluorescence intensity was measured using a SpectraMax i3 fluorometric microplate reader (Molecular Devices, LLC, USA) at excitation and emission wavelengths of 540 and 590 nm, respectively. Fluorescent images were captured using an EVOS FL fluorescence microscope (Life Technologies, CA, USA) following the protocol outlined in the MitoROS 580 kit.

#### 2.8.3. Analysis of Mitochondrial Membrane Potential

The JC-1 (J-aggregate-forming cationic dye) staining (Invitrogen, Thermo Fisher Scientific, USA) was used to measure the change in the mitochondrial membrane potential (ΔΨ) during apoptosis induced by the inhibitor treatment [39]. Cells were analyzed by acquiring 10,000 events using a BD FACSAria^TM^ III flow cytometer.

#### 2.8.4. TBARS Assay

The extent of lipid peroxidation was determined by measuring the level of malondialdehyde (MDA) formed using a thiobarbituric acid-reactive substances (TBARS) assay (Sigma-Aldrich, St. Louis, MO, USA) [40]. The TBARS assay was performed as previously described with minor modifications [41,42]. At the end of the experiment, the absorbance of the pink color (MDA-TBA adduct) was measured at 532 nm using a SpectraMax i3 microplate reader (Molecular Devices, LLC, San Jose, CA, USA).

#### 2.8.5. Wound Healing Assay

To assess the effect of single and dual treatments on the migration potency of HT-29 cells, an in vitro wound healing assay (scratch assay) was performed. Cells were plated, and the assay was conducted according to a previous study [43]. After creating the scratch, the medium was replaced, and the wells were washed to remove debris. Treatments were applied and the plates were incubated for 72 h at 37 °C in 5% CO_2_. Images were captured at 0, 24, 48, and 72 h by using a Nikon Eclipse inverted microscope (10× magnification). Image analysis was performed using the Fiji software (ImageJ version 2.9.0).

### 2.9. Statistical Analysis

The IC_50_ values for individual drugs were calculated using a non-linear regression model (four parameters) in GraphPad Prism 9.0. The dose–effect curves and combination index (CI) were assessed using the Chou–Talalay method [29], and CI plots were generated using ComboSyn 1.0 software (www.combosyn.com, accessed on 23 July 2022). CI values lower than 1 indicate synergism, values equal to 1 indicate additivity, and values greater than 1 indicate antagonism. Data are presented as mean ± standard error of the mean (SEM) of at least three independent replicates (n = 3) for each experiment with each CRC cell line. Differences between more than two groups were calculated using one-way analysis of variance (ANOVA) using GraphPad Prism version 9.0. A two-way analysis of variance (ANOVA) was used to compare the mean differences in the two variables between three or more groups. In all cases, *p*-values ≤ 0.05 were considered statistically significant.

## 3. Results

### 3.1. PRO and CAP Monotherapies Induced Cell Cytotoxicity in Cell Type-Specific and Dose-Dependent Manners

**Figure 1:**
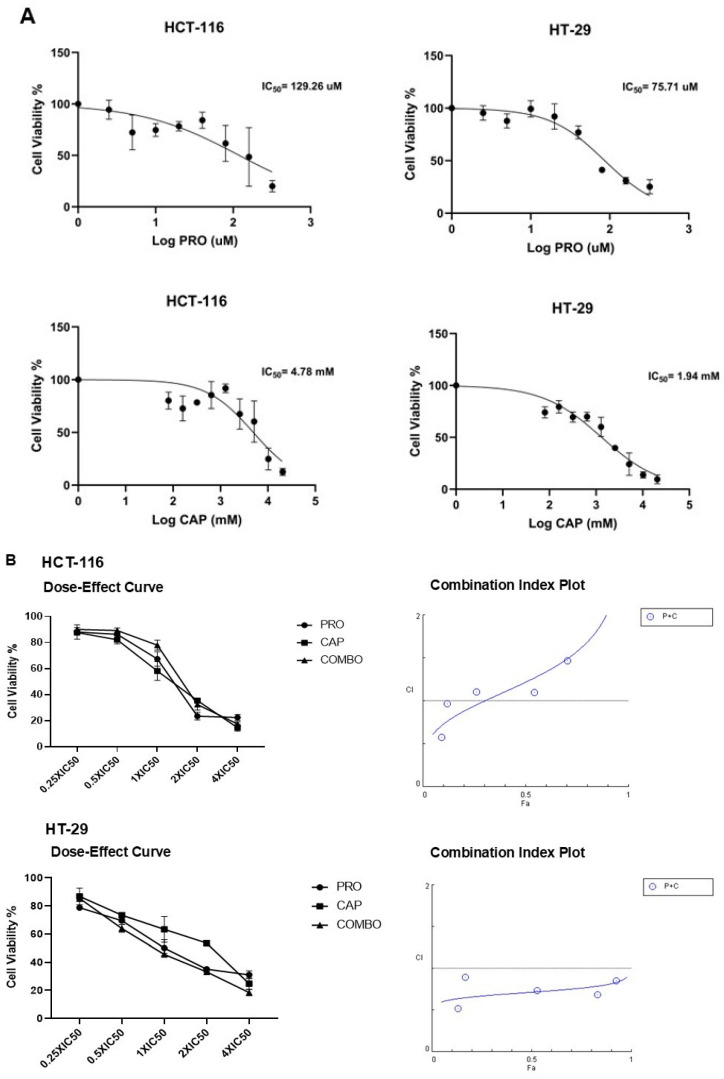

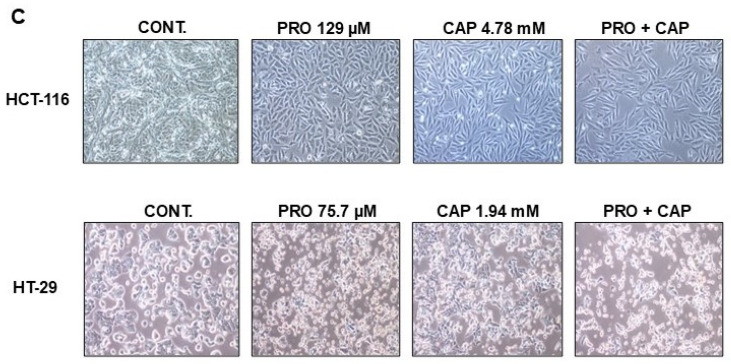
PRO and/or CAP inhibited the proliferation of CRC cell lines. (**A**) Representative dose–response curves of PRO and CAP monotherapy. (**B**) Drug combination study of double therapy on CRC cell lines. Histograms represent the dose–effect curves and combination index (CI) plots. Growth inhibition measurements were performed using MTT assay after 48 h incubation with a range of mono and combined drugs concentrations (0.25 × IC_50_, 0.5 × IC_50_, 1 × IC_50_, 2 × IC_50_, and 4 × IC_50_). CI values were calculated using ComboSyn software and histograms were obtained from ComboSyn software and GraphPad Prism. The results are expressed as the mean ± standard error. (**C**) Morphological images of CRC cells after PRO and/or CAP treatments at 10X magnification power. Microscopic images show the cytotoxic effects of the mono- and double-treatments on cells, such as the reduction in cell numbers, obvious alteration in the cell membranes, and the presence of apoptotic bodies in comparison to control cells.

The results of the MTT assay demonstrated that monotherapy decreased cell viability in a cell-type- and dose-dependent manner. Table 1 presents the IC_50_ values of the mono-treatments in the CRC cell lines. The dose–response curves of the effects of PRO and CAP monotherapies are shown in Figure 1A.

### 3.2. PRO Potentiates the Antiproliferative Effects of CAP in Cell Type-Specific Dependent Manner

The output of CompoSyn 1.0 software is shown in Figure 1B. Each cell line had a variable CI value, and thus different interactions with the combined treatments. The combined therapy displayed varied CI values depending on the cell type (Table 2)**.** Drug interaction in the HCT-116 cell line was additive, whereas a highly synergistic effect was observed in the HT-29 cell line. Moreover, the microscopic photographs (Figure 1C) showed obvious changes in the morphology of the combined-HT-29 treated cells when compared with untreated cells, which revealed the synergistic effects of PRO and CAP in HT-29 cells.

### 3.3. Effects of PRO and/or CAP Treatments on Induction of the Cellular Death Mechanisms

**Figure 2:**
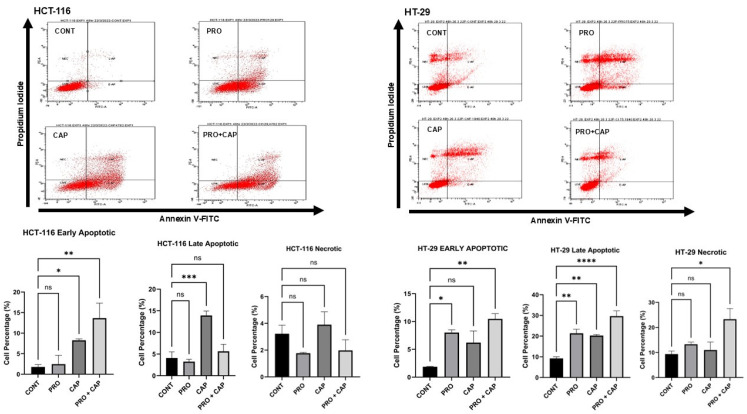
PRO and/or CAP-induced cell death in CRC cell lines. The four quadrants of the flow cytometry images were as follows: LIVE, non-stained cells (healthy cells); E-AP, early apoptotic cells conjugated with annexin V-FITC; L-AP, late apoptotic cells conjugated with annexin V-FITC and stained with PI; NEC = necrotic cells stained with PI. Values of early apoptotic, late apoptotic, and necrotic stages are expressed as the mean of three independent experiments ± standard error of the mean (n = 3 **±** SEM). Comparisons of means were made using a one-way ANOVA test using GraphPad Prism 9.5.1. ns = non-significant value (*p ˃* 0.05), * *p* ≤ 0.05, ** *p* ≤ 0.01, *** *p* ≤ 0.001, and **** *p* ≤ 0.0001. CONT, untreated cells.

Flow cytometry analysis revealed differences in the induction of cell death mechanisms between the treatments and the cell lines used. In the HCT-116 cell line (mut *PI3KCA* and mut *K-RAS*), the application of CAP and CAP + CAP significantly increased the percentage of cells in the early apoptotic stage compared to the untreated group, with no effect in the PRO group. The cell percentage at the early apoptotic stage was 8.25% and 13.65% for CAP and double groups, respectively, compared to the control (1.78%). Only CAP single treatment caused significant induction of apoptosis in the late stage of these cells, with a percentage of 13.93% versus control (4.10%) (Figure 2). For the HT-29 cell line (mut *PI3KCA*, mut *TP53*, and mut *B-RAF*), the application of PRO- and double treatments significantly increased the percentage of cells in the early apoptotic stage compared to the control, with no effect within the CAP group. The cell percentages at the early apoptotic stage were 8.00% and 10.47% for PRO and double groups, respectively, compared to the control (1.86%). All treatment groups increased the cell percentage at the late apoptotic stage, with a high level of significance in the double group (PRO = 21.33%, CAP = 20.23%, and double group = 29.68% vs. 9.20% for control). Moreover, the overall data from the double group showed that necroptotic cell death was induced in HT-29 cells (Figure 2). The results demonstrated the synergistic effect of double therapy in the HT-29 cell line through the induction of cell death at significantly higher levels than monotherapy in comparison to the control. However, the overall data for the combined treatment did not show a synergistic effect in the HCT-116 cell line, which may be related to the genetic background.

### 3.4. Effects of PRO and/or CAP Treatments on Transcriptomic Profile of HT-29 Cell Line

#### 3.4.1. Overall View of the Transcriptome Profile

**Figure 3:**
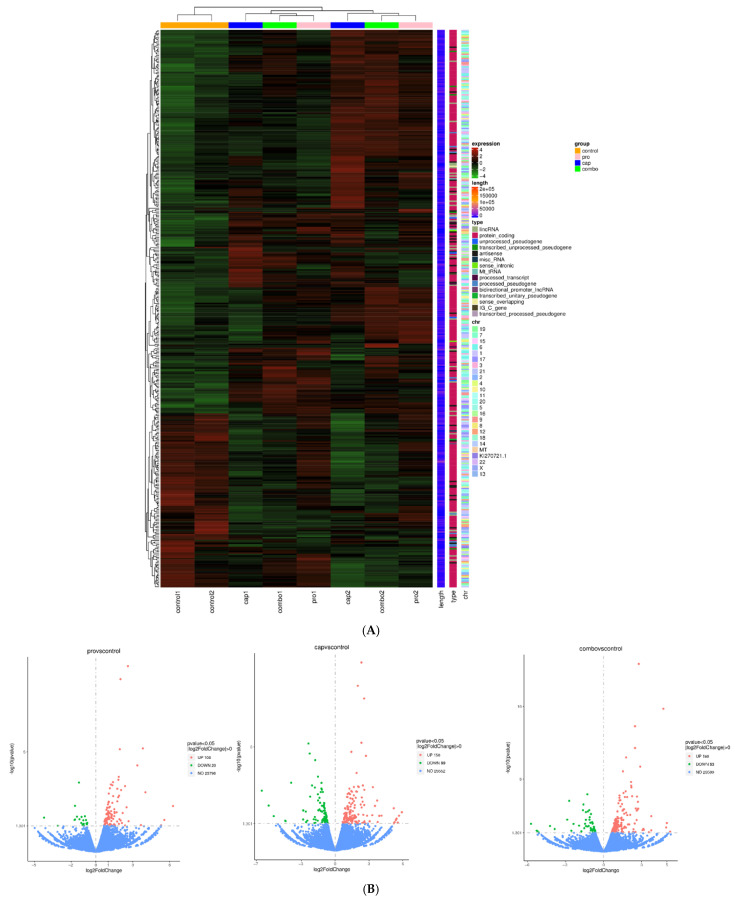

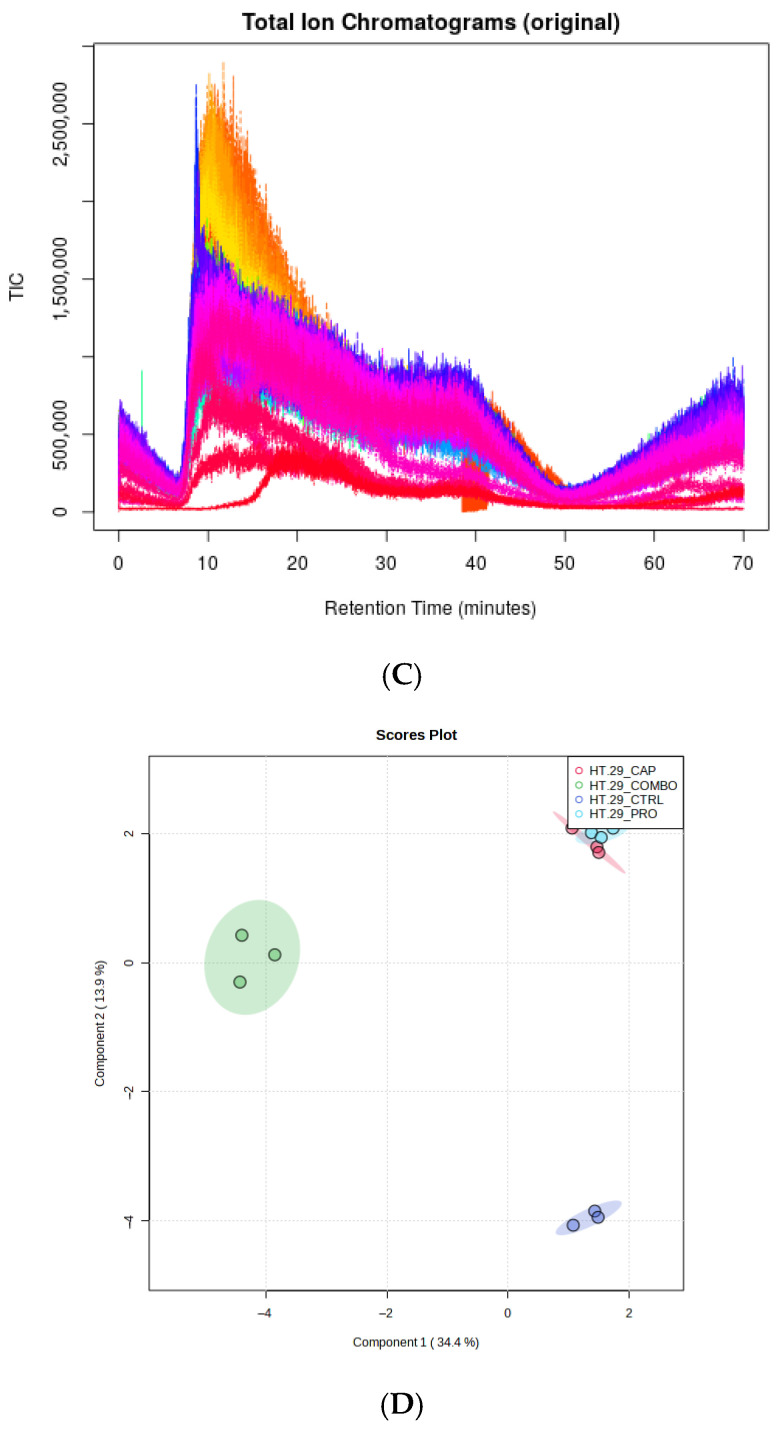

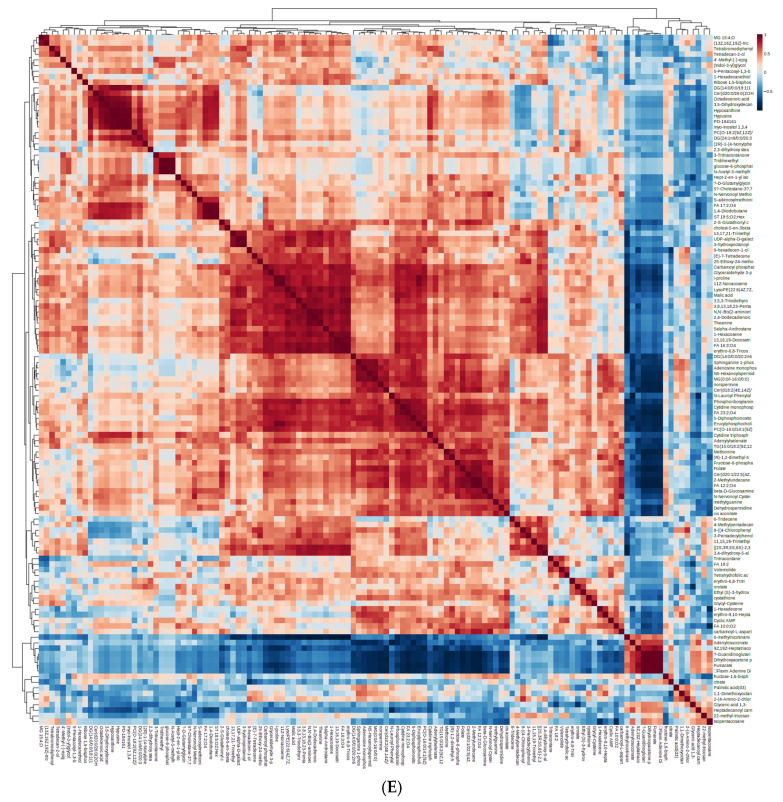

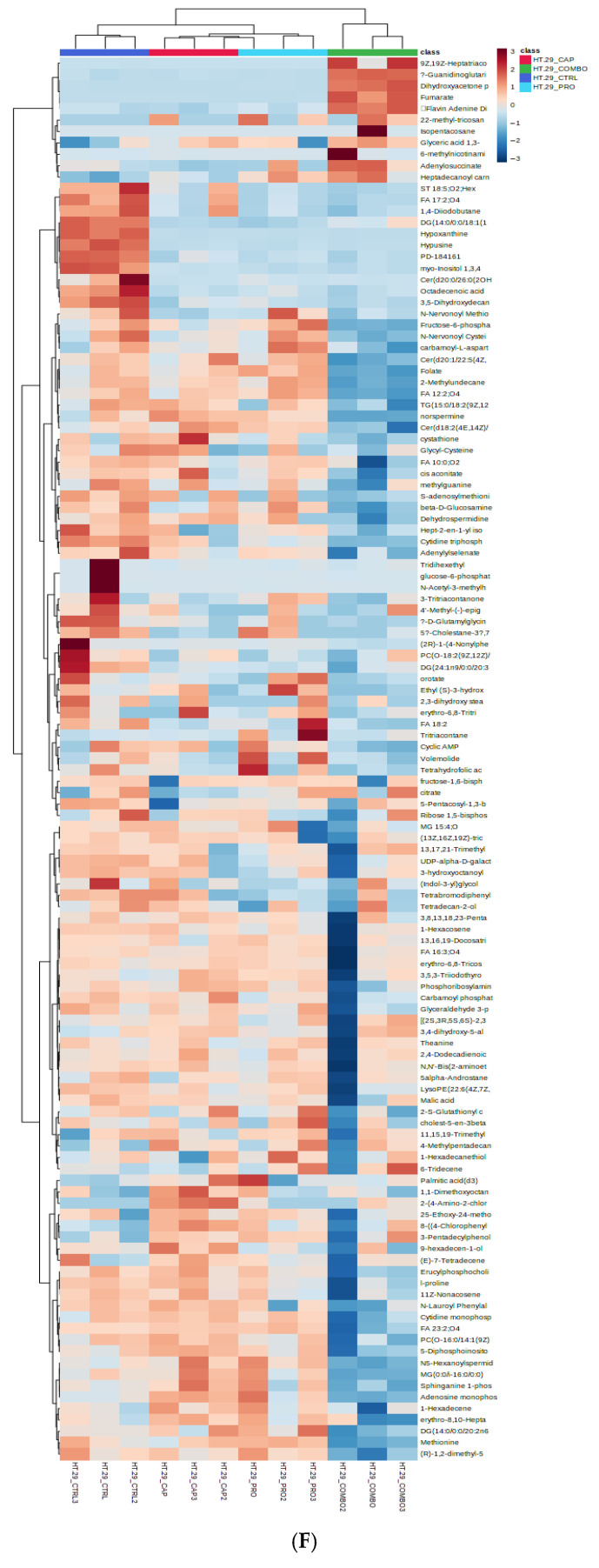

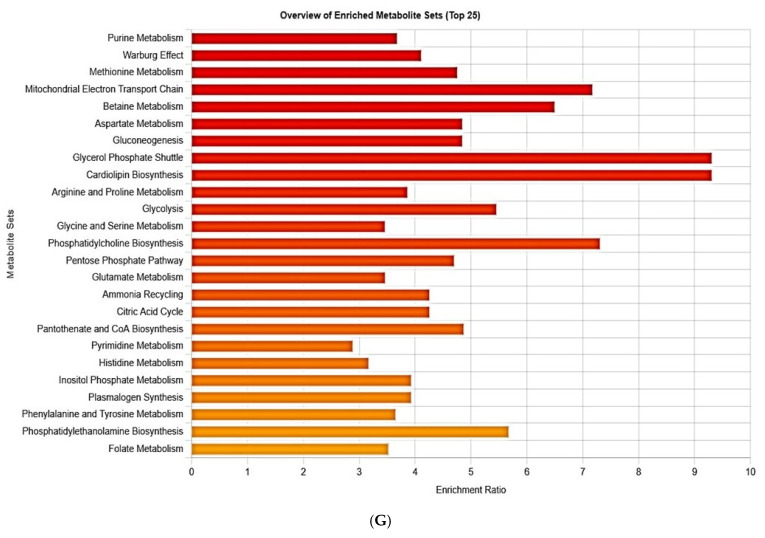
Overall view of the transcriptomic and metabolomic profiles of HT-29 cells after treatment with PRO and/or CAP compared with untreated cells. (**A**) Heatmap showing the expression pattern of genes in HT-29-treated groups versus the control group. (**B**) Volcano plot showing the positions of differentially upregulated and downregulated genes in the PRO, CAP, and PRO + CAP groups versus the control group. (**C**) Total ion chromatograms of the extracted metabolites from treated and untreated HT-29 cells, which were run in LTQ-XL linear ion trap LC-MS. (**D**) Principal component analysis (PCA) of comprehensive metabolites from treated and untreated HT-29 cells. (**E**) Correlation heatmaps of treated and untreated HT-29 cells. (**F**) Heatmaps of differentially expressed metabolites in treated and untreated HT-29 cells. (**G**) The top twenty-five pathways enriched in the metabolome analysis of treated and untreated HT-29 cell lines.

High-throughput sequencing of messenger RNA (mRNA-seq) for the control and treated colorectal adenocarcinoma HT-29 cells was conducted using Novogene (https://www.novogene.com/us-en/, accessed on 23 July 2022). Samples from both treated (PRO and/or CAP) and untreated cells were used for mRNA-seq, and the results revealed interesting expression patterns. As shown in Figure 3A, the heatmap implies that mono- and dual treatments have strong effects on HT-29 cells, resulting in transcriptomic variation after treatment with PRO, CAP, and PRO + CAP compared to the control.

#### 3.4.2. Identification of DEGs

Differential expression analysis of the acquired genes was performed using the DESeq2 R package (1.20.0) to determine the DEGs between the two groups, with an adjusted *p*-value of ≤0.05. Differential expression analysis identified 126 differentially expressed genes (DEGs) that were significant in the high-throughput RNA sequencing of HT-29 cells treated with PRO monotherapy, including 106 differentially expressed upregulated genes (DUGs) (padj < 0.01, log2 FC > 2) and 20 differentially expressed downregulated genes (DDGs) (padj < 0.01, log2 FC < −2), which were obtained in this study. The analysis identified 257 DEGs that were significant in the high-throughput RNA sequencing of HT-29 cells treated with CAP monotherapy, including 158 DUGs (padj < 0.01, log2 FC > 2) and 99 DDGs (padj < 0.01, log2 FC < −2), which were obtained in this study. For dual therapy (PRO plus CAP), the analysis identified 213 DEGs that were significant in the high-throughput RNA sequencing of HT-29 cells treated with CAP monotherapy, including 160 DUGs (padj < 0.01, log2 FC > 2) and 53 DDGs (padj < 0.01, log2 FC < −2), which were obtained in this study.

The volcano plot in Figure 3B presents a summary view of significant DEG counts according to fold change and significance elicited by all HT-29 treated cells with PRO and/or CAP versus control cells.

In Supplementary Materials Table S1, we also present the top 50 significant DEGs in HT-29 cells after treatment with PRO, CAP, and PRO + CAP compared to the control. These data files included gene ID, average Log_2_ (signals) fold change (LFC), *p*-value, *p*-adjusted value (padj), gene symbol, and gene chromosome for the single treatments (PRO or CAP samples), and combination treatment (PRO plus CAP samples) versus control samples.

### 3.5. Effects of PRO and/or CAP Treatments on Cellular Metabolome Profile

#### Overall View of Metabolomic Profiles

HPLC-MS/MS spectral separation (TIC, total ion chromatograms) of the metabolites is shown in Figure 3C. A wide-range, extensive metabolite list with identification, features, peak intensity values, and *p*-values was obtained from the analysis. The HMDB database with ESI positive-mode analysis was used to identify metabolic markers. From a total of 144 metabolites that were detected in the control and treated cells, only 123 metabolites were employed for further analysis, owing to the subtraction of duplicate compounds identified across replicates. Using MetaboAnalyst 5.0, only thirty-five metabolites from 123 features in the HT-29 cell line showed a significant difference (*p* ≤ 0.05), as calculated by one-way ANOVA and post-hoc analysis (Supplementary Materials Figure S1).

The overall correlation coefficient heatmaps of the metabolomics data illustrated self-correlations between the metabolites (Figure 3E). As presented in Figure 3F, the heatmaps for the comprehensive metabolites demonstrated variation in metabolic accumulation among the examined HT-29 cell lines for the control, PRO, CAP, and combined groups.

These results indicated that PRO and/or CAP treatment altered the metabolic phenotype of HT-29 cells in different manners and displayed a differential metabolic response. Moreover, evidence based on metabolic variation in the HT-29 cell line suggests unique metabolomic profiles for treated and untreated cells (Figure 3F).

Principal component analysis, as shown in Figure 3D, demonstrated four metabolic clusters composed of the untreated HT-29 group and the PRO and/or CAP groups. The plot showed a clear difference in metabolomics between control and cells treated with dual therapy, while metabolic clusters of monotherapy groups were close to each other and, at the same time, far from the combined and control groups.

Additionally, enrichment and pathway analyses revealed that the enriched metabolic pathways were eighty-two. The analysis showed alterations in many enriched metabolic pathways, including glutamate metabolism, methionine metabolism, TCA cycle, mitochondrial electron transport chain, and phosphatidylcholine biosynthesis. The top significant twenty-five enriched metabolic pathways are shown in Figure 3G.

### 3.6. The Combination Treatment Induces the Ferroptosis in HT-29 Cells

#### 3.6.1. Ferroptosis Pathway

**Figure 4:**
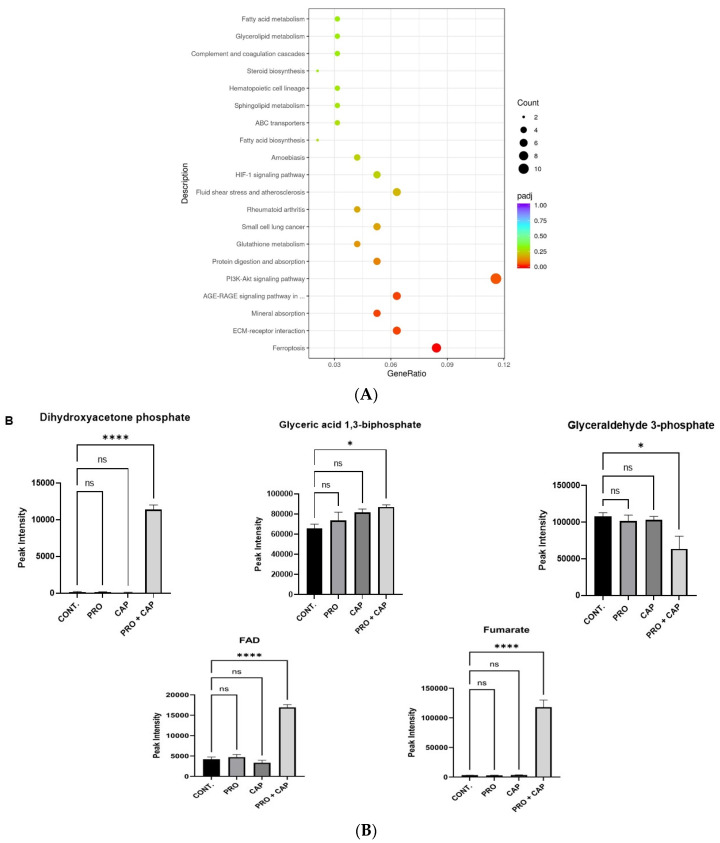

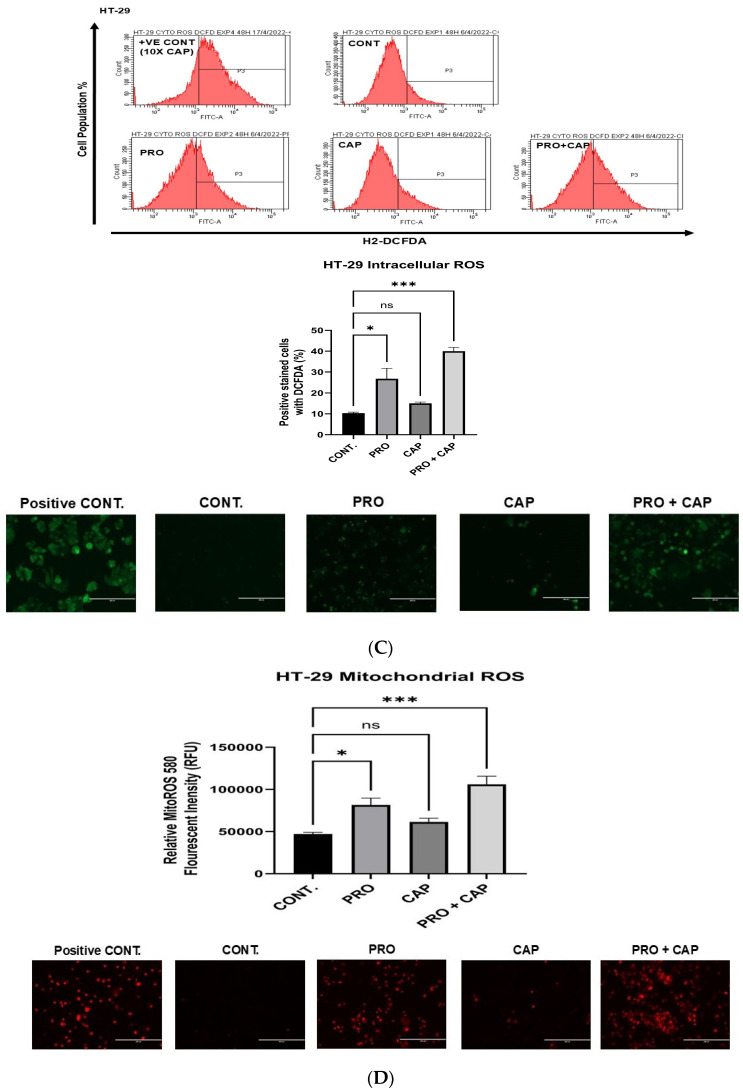

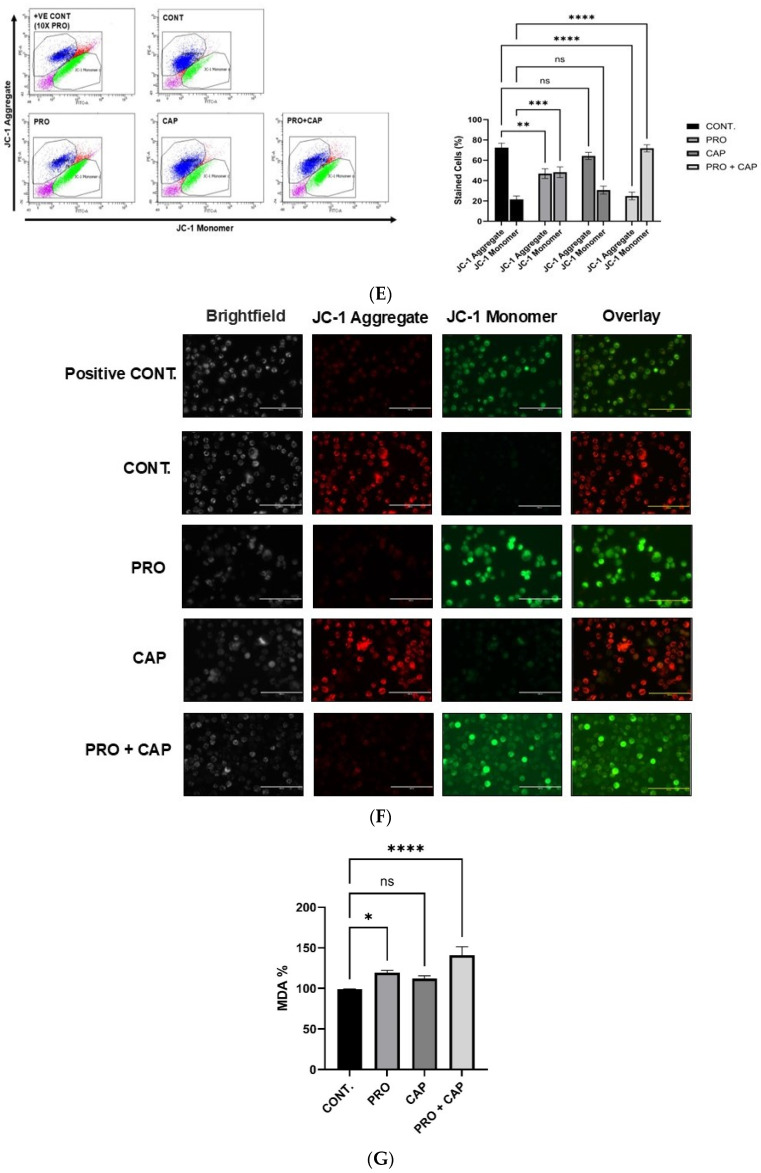
The dual treatment induces ferroptosis in HT-29 cells. (**A**) KEGG pathway enrichment analysis for the most significantly enriched pathways in HT-29 cells treated with PRO + CAP. (**B**) Histograms showing the quantitative levels of various metabolites involved in the OXPHOS pathway in the treated and untreated HT-29 cell line. (**C**) Measurement of intracellular ROS levels. Images were captured at a magnification power of 20×. +VE CONT, positive control group treated with 10× CAP. (**D**) Generation of mitochondrial ROS. All images were captured at a magnification power of 20× using an EVOS FL. (**E**) Changes in mitochondrial membrane potential. (**F**) The fluorescent images of mitochondrial membrane potential changes in HT-29 cells after treatment with PRO and/or CAP for 48 h using JC-1 dye. Images were captured at a magnification power of 40×. The bright field indicates the images with no color, JC-1 Aggregate indicates the images of mitochondria that stained with red fluorescence color (polarized mitochondrial membrane), JC-1 monomer indicates the images of mitochondria that stained with green fluorescence color (depolarized mitochondrial membrane), and the overlay shows the merge between red and green fluorescence colors that were acquired using Fiji software (ImageJ version 2.9.0) (https://imagej.net/software/fiji/). +VE CONT, positive control group treated with 10× PRO. (**G**) Quantitative analysis of MDA levels in HT-29 cells treated with PRO and/or CAP. The data are expressed as mean of three independent experiments ± standard error of the mean (n = 3 ± SEM). Comparisons of means were made using one or two-way ANOVA tests in GraphPad Prism 9.5.1 software. ns = non-significant value (*p* ˃ 0.05), * *p* ≤ 0.05, ** *p* ≤ 0.01, *** *p* ≤ 0.001, and **** *p* ≤ 0.0001. CONT, untreated cells.

Interestingly, KEGG pathway enrichment analysis demonstrated that ferroptosis (ID: hsa04216) (Figure 4A) was enriched at a highly significant level (*p*-value = 6.79 × 10^−8^ versus control) for the dual treatment (PRO plus CAP) than in other single treatments (*p*-value = 0.05; PRO, *p*-value = 3.34 × 10^−6^ for CAP versus control), which confirmed the synergistic interaction of the dual treatment that was obtained from the cytotoxicity study. In addition, the number of significantly enriched genes related to ferroptosis was eight genes for dual treatment, six genes for CAP, and two genes for PRO (Table 3). This finding revealed a synergistic effect when both PRO and CAP were applied to the HT-29 cells.

Moreover, KEGG pathway enrichment analysis showed that another cell death mechanism, necroptosis, was activated during the application of the double treatment (Supplementary Materials Table S2). This pathway was upregulated by increasing the transcript levels of five genes: SQSTM1, FTH1P2, FTH1P8, FTH1P23, and FTH1. In line with this, flow cytometry analysis of the combination group in HT-29 cells showed activation of necroptosis compared to the control (Figure 2). These findings revealed that the double treatment induced ferroptosis coupled with necroptosis in HT-29 cells.

The activation of oxidative metabolism (OXPHOS), over-accumulation of ROS, reduced MMP, and high MDA levels are features of ferroptosis upregulation [44,45,46]. To gain validated evidence for the induction of ferroptosis in HT-29 cells after the treatments, we further conducted untargeted metabolomic profiling and performed different biochemical assays, including assessments of ROS generation, MMP analysis, and lipid peroxide assays.

#### 3.6.2. Mitochondrial Oxidative Metabolism (OXPHOS)

OXPHOS is a mitochondrial bioenergetic molecule known as the mitochondrial electron transport chain (METC). Additionally, METC acts as a major source of ATP production and Mito ROS generation; hence, it serves as a targeting strategy for cancer therapy [47]. OXPHOS plays a principal role in promoting ferroptosis [46]. Therefore, we examined the accumulation levels of metabolites related to OXPHOS in HT-29 cells after treatment compared to those in control cells. The intermediate metabolites of OXPHOS in the combined group of HT-29 cells, such as flavin adenine dinucleotide (FAD), fumarate, dihydroxyacetone phosphate (DHAPH), and glyceric acid-1,3-bisphosphate, were elevated to a greater extent (Table 4 and Figure 4B). This metabolic result confirmed that OXPHOS promoted ferroptosis in the combined group of HT-29 cells.

#### 3.6.3. Oxidative Stress-Related Genes

The analysis of DEGs’ double treatment demonstrated a significant upregulation of the transcript of the OSGIN1 gene, oxidative stress-induced growth inhibitor 1 gene (*p*-value = 0.002 versus control). Moreover, we observed a decline in LFC of GPX4 and glutathione synthetase (GSS) transcripts in the combined group (LFC GPX4 = −0.18 and LFC GSS = −0.08 versus control).

#### 3.6.4. Generation of Intracellular Reactive Oxygen Species (ROS)

The data indicated that the level of intracellular ROS in the HT-29 cell line increased with PRO treatment, but a marked increase was observed with co-treatment versus the control, and no change was observed with CAP treatment (Figure 4C). This revealed that PRO sensitized the CAP HT-29 cell line (synergistic addition) to intracellular ROS generation. The percentages of ROS levels were 26.80, 15.06, and 40.03% for PRO, CAP, and co-treatment versus control (10.35%).

#### 3.6.5. Generation of Mitochondrial Reactive Oxygen Species (Mito ROS)

As shown in Figure 4D, the analysis determined that mono PRO increased Mito ROS (* *p* ≤ 0.05), but the combination therapy dramatically elevated the amount of Mito ROS (*** *p* ≤ 0.001) compared to the control, and no fold change was detected with CAP alone. Moreover, the results suggest that the overgeneration of Mito ROS in the combined group of HT-29 cells is a consequence of OXPHOS upregulation, which further promotes ferroptosis.

#### 3.6.6. The Analysis of Mitochondrial Membrane Potential

The data showed that both PRO and combined treatments reduced the polarization of mitochondrial membrane potential in HT-29 cells, as evidenced by the JC-1 dye. Among all treatment groups, only PRO- and combined treated cells showed a significantly greater JC-1 monomer population (depolarized mitochondrial membrane with green fluorescent color) with percentages of 48.30% and 71.80%, respectively, compared to untreated cells (21.53%), whereas JC-1 aggregate percentages (polarized mitochondrial membrane with red fluorescent color) were significantly diminished to 46.86% and 24.90% for the previously mentioned treatments compared to control cells, which had high contents of polarized mitochondrial membranes (72.43%). However, CAP did not alter the polarization of the mitochondrial membrane (Figure 4E). Moreover, the examination of the fluorescence images in Figure 4E shows the consistency of the fluorescence images with the flow cytometry data, as described above.

#### 3.6.7. Lipid Peroxidation Level

The analysis showed that combined treatment significantly increased the MDA levels to 140.66% greater than PRO in comparison to control cells, while CAP treatment resulted in a relative increase in MDA but did not reach a significant level in contrast to the control (Figure 4G). Finally, the cumulative data from previous experiments further confirmed the synergistic induction of ferroptosis in the combined group of HT-29 colorectal adenocarcinoma cells.

### 3.7. The Combination Treatment Inhibits the Cell Migration of HT-29 Cells

**Figure 5:**
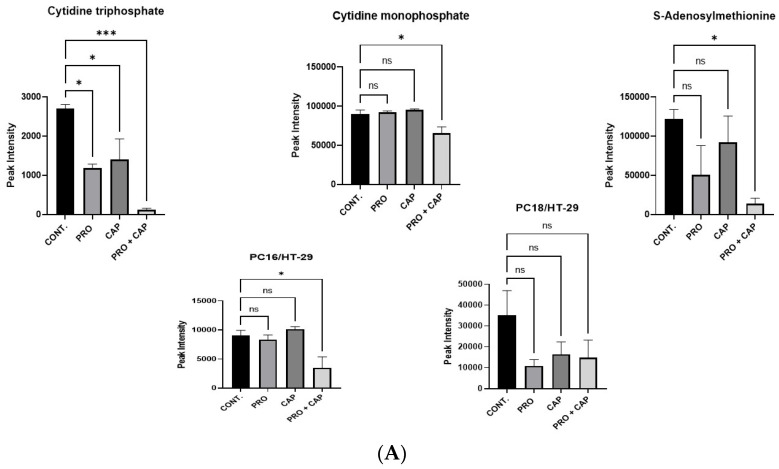

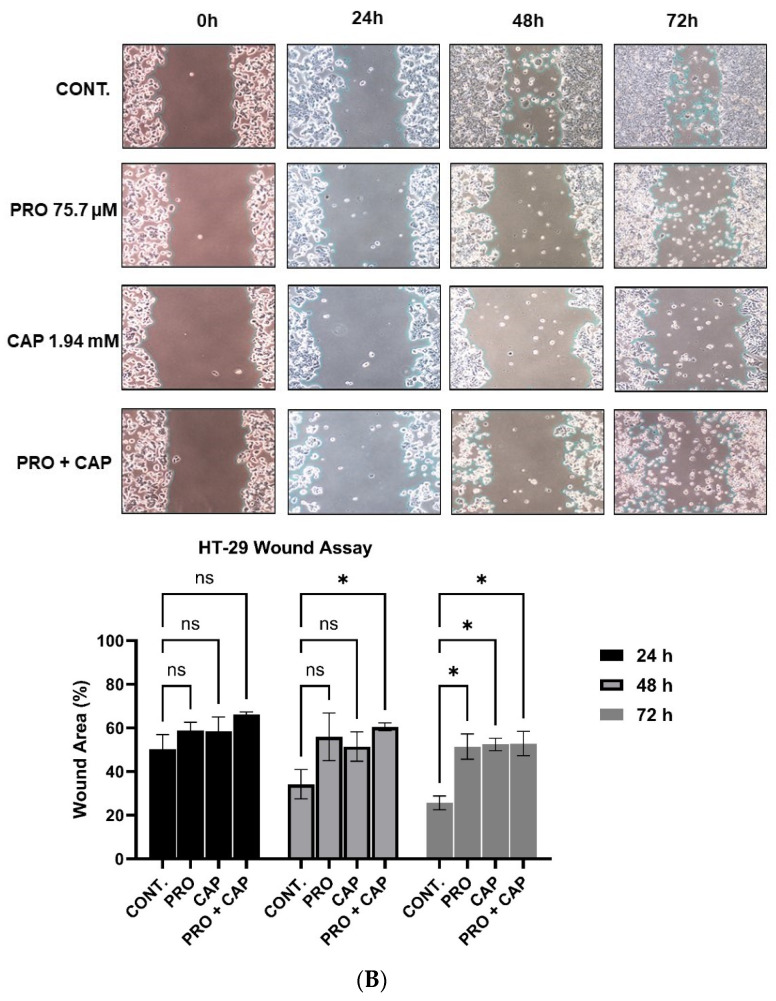

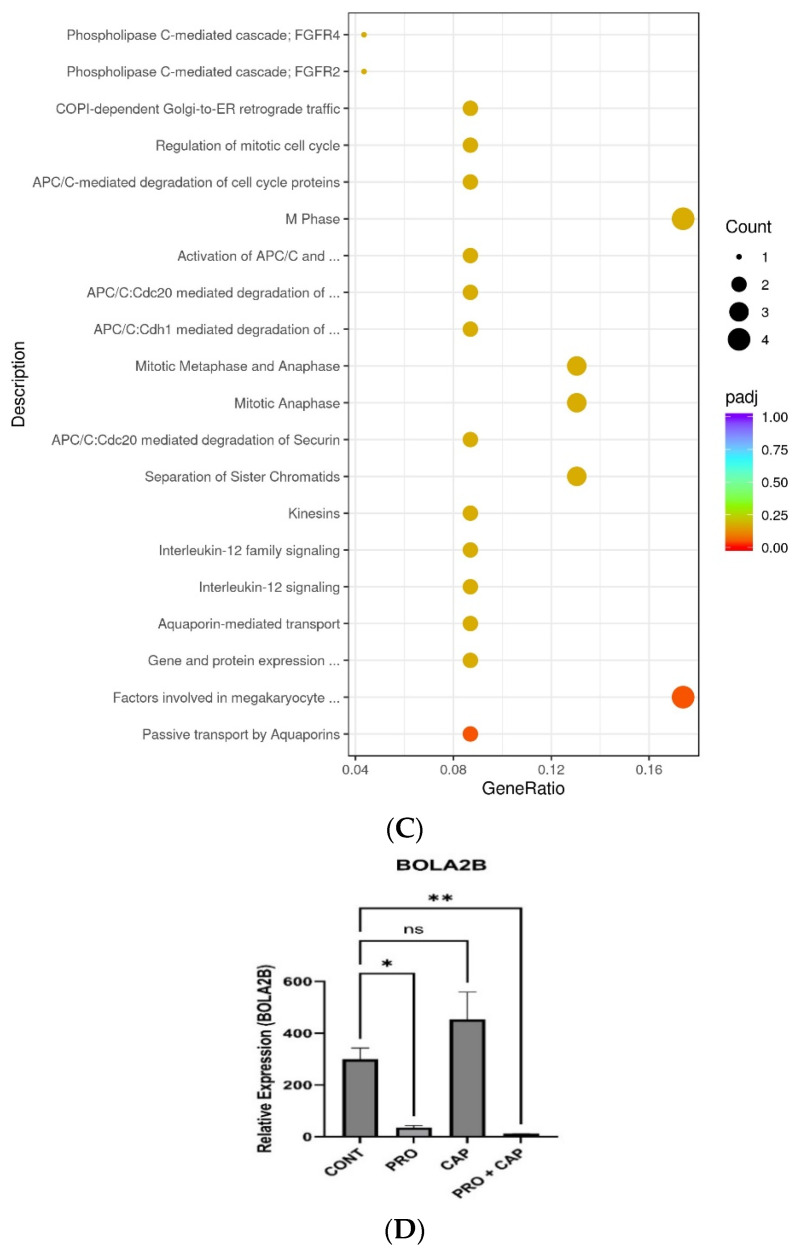
The dual treatment inhibits cell migration and triggers the immune response in HT-29 cells. (**A**) Histograms showing the quantitative levels of various metabolites involved in phosphatidylcholine biosynthesis in the treated and untreated HT-29 cell line. (**B**) Representative images of the wound healing assay for HT-29 cell line after treatment with PRO and/or CAP. Histograms represent the differences in gap area % of treated groups versus control over time. (**C**) Reactome pathway enrichment analysis of the most downregulated pathways in HT-29 cells treated with PRO + CAP showing the pathway of gene and protein expression by JAK-STAT signaling after interleukin-12 stimulation. (**D**) The mRNA relative expression of *BOLA2B* gene in HT-29 treated with combined group. The data are expressed as mean of three independent experiments ± standard error of the mean (n = 3 ± SEM). Comparisons of means were made using one or two-way ANOVA tests in GraphPad Prism 9.5.1 software. ns = non-significant value (*p* ˃ 0.05), * *p* ≤ 0.05, ** *p* ≤ 0.01, and *** *p* ≤ 0.001. CONT, untreated cells.

Analysis of metabolomic data revealed an alteration in phosphatidylcholine biosynthesis in the combined group of HT-29 cells. Phosphatidylcholine (PC) is a principal component of cellular phospholipids and it plays a critical role in the cell membrane structure and signaling in all mammalian cells. Loss of cell membrane integrity and inhibition of the choline pathway disrupts PC homeostasis, leading to growth arrest or cell death [48]. In addition, a decrease in PC is linked to susceptibility to ferroptosis [49]. Moreover, it was reported that inhibition of phosphatidylcholine metabolism leads to a decrease in the migration and invasion potential of breast cancer cells [50]. In the current study, the addition of combined treatments to HT-29 cells induced cell death (ferroptosis), thereby causing a reduction in PC metabolites such as cytosine triphosphate (CTP), cytosine monophosphate (CMP), SAM, and PC (Table 4 and Figure 5A). Hence, previous data have suggested that such effects could be a consequence of cell migration inhibition and ferroptosis induction.

Furthermore, we carried out an in vitro wound healing assay to assess the cell migration potency of HT-29 cells after treatment. Processed images were acquired to highlight the migration of HT-29 cells after treatment and over time. The co-treatment group of HT-29 cells showed a significant reduction in cell migration capacity at 48 and 72 h, whereas the monotherapies showed a significant reduction in cell migration capacity only at 72 h (Figure 5B). The results showed a synergistic anti-migration effect of PRO coupled with CAP on HT-29 cells.

### 3.8. The Combination Treatment Triggers the Immune Response in HT-29 Cells via Blocking the JAK-STAT Signaling

**Figure 6:**
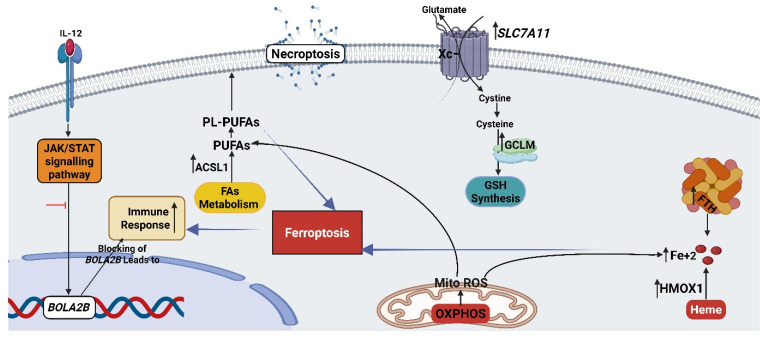
Proposed mechanism of combined therapy in HT-29 cell line based on RNA-seq results. The figure was created with BioRender (www.biorender.com).

Reactome pathway enrichment analysis demonstrated that PRO and combined treatments caused the inhibition of gene and protein expression by JAK-STAT signaling after interleukin-12 stimulation in HT-29 cells via a significant reduction in *BOLA2B/LOC107984053* gene transcripts compared to untreated cells (Figure 5C,D) (Supplementary Materials Tables S2 and S3). Moreover, the degree of inhibition of JAK-STAT signaling in the combined group (*p*-value _PRO+CAP_ = 0.004 versus the control) was greater than that of PRO alone (*p*-value = 0.01 versus control), which supports the benefits of using the double combination therapy approach in treating BRAF^V600E^-mutant mt CRC. In addition, this finding revealed that the combined treatment induces an immune response in HT-29 cells through the inhibition of JAK-STAT signaling after interleukin-12 stimulation, Figure 6.

## 4. Discussion

This study aimed to explore uncharted territory in the field of cancer therapy by investigating the potential anti-cancer effects of PRO and CAP as monotherapy and dual therapy across human CRC cell lines (HCT-116 and HT-29).

The MTT assay results indicated that the administration of PRO or CAP as individual treatments reduced the proliferation of human CRC cell lines in a cell type-specific and concentration-dependent manner, which is in agreement with previous studies [11,51,52,53,54,55], depending on the cell type, dosage, culture conditions, and time duration. Accordingly, the current data demonstrated that HCT-116 had a high IC_50_ for PRO, whereas HT-29 had the lowest IC_50_. HCT-116 and HT-29 cells had a mutation in the *PIK3CA* gene, but HT-29 cells were more sensitive to PRO than HCT-116 cells. Our in vitro study revealed for the first time that PRO can potentiate the antiproliferative effects of CAP in a cell type-specific manner. Moreover, HT-29 cells were among the most responsive to the combination (PRO + CAP), even in the presence of *PIK3CA*, *B-RAF* (V600E), and *TP53* mutations. The varied response of the cells to the treatment might be due to the variation in their features, as well as other potentially involved genetic and epigenetic factors mentioned in Supplementary Materials Table S4.

In vitro studies have examined the combination of PRO with different chemotherapeutic agents (5-FU, paclitaxel, or cisplatin), and their results showed that PRO could modulate the antiproliferative effects of these agents in a cell type-specific, chemotherapeutic drug-dependent, and concentration-dependent manners. The combined therapy results in synergistic, additive, or antagonistic effects in normal human and cancer cell lines [55,56]. The previous findings agree with our results that the effects of the combination of PRO and CAP ranged from synergism and additive, depending on the type of cell line, dose, and time point.

Furthermore, flow cytometric analysis of cell death in combined groups of HCT-116 and HT-29 cells revealed a synergistic effect of the addition of PRO to CAP in HT-29 cells. However, there is no evidence in the literature regarding PRO plus CAP treatment. Moreover, there are few reports on PRO and CAP as monotherapies, demonstrating that these single agents could induce apoptosis in different cell types, including CRC cells, depending on the cell type, dosage, culture conditions, and time duration [54,57,58,59].

It is well known that colorectal tumorigenesis is initiated by the accumulation of genetic and epigenetic alterations that can dysregulate cellular metabolism in CRC [3]. This metabolic reprogramming of CRC is characterized by unique metabolic phenotypes identified in the literature [3,5,60].

As PRO + CAP treatment showed a synergistic effect on HT-29 cells, we further investigated the underlying molecular mechanisms of this treatment. In this study, we profiled the cellular metabolome and transcriptome of the HT-29 colorectal adenocarcinoma cell line as a CRC preclinical model (in vitro) after treatment to identify novel targets involving cellular metabolites and transcripts. Accordingly, we identified novel metabolites, transcripts, and critical regulatory pathways that were affected by the co-treatment.

Molecular profiling technologies, including transcriptomics and metabolomics, have become essential tools for gaining a more detailed and systematic understanding of drug actions, particularly in the context of anti-cancer drugs. This signature-based approach to drug characterization offers a comprehensive molecular and phenotypic description of the cellular processes and pathways affected by the drug, thereby defining its broader mode of action [61]. In our study, transcriptomic profiling of HT-29 cells demonstrated the synergistic induction of the ferroptosis pathway in the co-treated group. Ferroptosis, a novel form of programmed cell death (PCD), relies on iron-dependent lipid ROS and is recognized as an oxidative cell death process [44,62,63]. Unlike apoptosis and other well-known forms of cell death, ferroptosis has distinct genetic, immune, biochemical, and morphological characteristics [63]. Ferroptosis involves three distinct classes of metabolic pathways and mechanisms (canonical ferroptosis) and other metabolic pathways (non-canonical ferroptosis) [44,63]. The first class is ferroptosis, which is iron metabolism-dependent. Second, ferroptosis is dependent on lipid metabolism. Third, ferroptosis is dependent on system Xc^−^/GSH synthesis/GPX4 [44,64]. Genetically, the underlying mechanism of each pathway is regulated through multiple genes/proteins that do not occur independently but influence each other [44]. Biochemically, it is accompanied by an overload of redox-active iron (Fe^+2^), aberrant ROS generation, GSH depletion, and excessive lipid oxidation of polyunsaturated fatty acids (PUFA) [44,45,65]. Ferroptosis has a dual role in cancer. It plays a role in tumorigenesis and a therapeutic role in cancer [63].

Interestingly, co-therapy drives HT-29 cells toward ferroptosis with a high degree of significance and a greater number of enriched genes (eight) than that observed with monotherapy. Here, ferroptosis induction resulted in the upregulation of *HMOX1*, *FTH1*, *FTH1P2*, *FTH1P8*, *FTH1P23*, *ACSL1*, *SLC7A11*, and *GCLM* transcripts. The latter gene, *GCLM*, participated in the glutathione synthesis. Hence, increased expression of *SLC7A11* and *GCLM* may be involved in the combined treatment-induced ferroptosis in HT-29 cells, which is thought to be initiated in response to the rapid depletion of GSH and the huge ROS burden after treatment [66,67]. In addition, enrichment of the *ACSL1* transcript instead of *ACSL4* revealed that this isoform also plays an important role in ferroptosis induction, which agrees with a recent study [68]. In brief, among the ferroptosis mechanisms, lipid metabolism has a major role in driving ferroptosis [65]. Moreover, the ferroptotic activity of co-therapy in HT-29 cells is triggered by three classes of ferroptosis pathways, which reflects the synergistic effect. The strength of ferroptosis in the co-therapy group is also manifested by the inclusion of certain genes that have a high capacity of function in the ferroptosis pathway through the upregulation of FTH1 [69] and SLC7A11 [44,66]. Furthermore, a recent study has reported that increased levels of SLC7A11 are involved in ferroptosis induction [70].

Furthermore, RNA-seq analysis showed that increased transcript levels of genes linked to ferroptotic iron metabolism (*FTH1P2*, *FTH1P8*, *FTH1P23*, and *FTH1* genes) were enriched in the necroptosis pathway with increased levels of *SQSTM1* transcripts. This reveals the contribution of these mechanisms (necroptosis overlaps with ferroptosis). Moreover, upregulation of SQSTM1 participates in necroptosis [71] and ferroptosis [72]. Biochemically, this was observed in our study through necroptosis induction, which was determined by annexin-V/PI flow cytometric analysis in the double group of HT-29 cells (Figure 2). In addition, metabolomic data demonstrated the disruption of PC content in the cell membrane through the induction of ferroptosis/necroptosis.

Moreover, cumulative transcriptomic and metabolomic analyses showed that the upregulation of ferroptosis in the co-therapy group of HT-29 cells occurred via canonical and non-canonical pathways. The latter is stimulated via a mechanism that is linked to the mitochondrial pathway called OXPHOS (METC) [44,64]. Recently, OXPHOS was found to promote ferroptosis [46].

The metabolic fingerprint after treatment demonstrated that the OXPHOS pathway was upregulated to a greater extent upon co-treatment in HT-29 cells. OXPHOS is a mitochondrial metabolic pathway that generates mitochondrial ROS [73]. This effect was demonstrated by the generation of highly significant levels of both intracellular and mitochondrial ROS in the combined group. From this perspective, we hypothesized that a highly significant level of both cytoplasmic and mitochondrial ROS could be a result of the leakage of mitochondrial ROS into the cytoplasmic pool, thereby collectively elevating cellular ROS. This could be caused by a decrease in mitochondrial membrane potential, which permits the release of ROS, as shown in this study.

Recently, it was shown that the augmentation of ROS production could be the key to opening a new door in tumor inhibition [74]. Importantly, excessive ROS generation is a proven cellular marker of lethal lipid peroxidation that results in cell death [44,66]. These findings strongly indicate that double treatment might cause ROS-induced lethal oxidative damage (ferroptosis) in HT-29 colorectal adenocarcinoma cells.

Furthermore, the metabolic data demonstrated a decrease in phosphatidylcholine biosynthesis that facilitated the inhibition of migratory potency in the HT-29-co-treated group and the induction of ferroptosis, which agrees with previous research [49,50].

In addition, the collective analysis of several biochemical parameters showed a significant over-accumulation of intracellular and Mito ROS, upregulation of the OXPHOS (METC) metabolic pathway, disruption in phosphatidylcholine (PC) biosynthesis, reduction in MMP, and high MDA levels induced by combined treatment, which are hallmarks of ferroptosis [44,45,49,63,65,75]. Malondialdehyde (MDA) is known as one of the biochemical features of ferroptosis activation. It is a product of lipid peroxidation that destroys cell membrane integrity during ferroptosis [64]. In the combined group, upregulation of the *ACSL1* gene increases the polyunsaturated fatty acid (PUFAs) content in phospholipids, which then become highly susceptible to oxidation reactions by ROS, finally leading to ferroptosis [64]. Moreover, the upregulation of iron metabolism leads to the accumulation of iron ions (Fe^+2^). These Fe^+2^ ions may undergo the Fenton reaction, which is iron-mediated ROS production, thereby promoting lipid peroxidation (high MDA level) that activates ferroptosis [62].

Furthermore, *OSGIN1*, known as a tumor suppressor gene that is linked to mitochondrial ROS and then ferroptosis induction, was upregulated, which suggests that the addition of PRO to CAP stimulated the oxidative stress, elevating Mito ROS and induced ferroptosis in HT-29 cells [76]. In contrast, the decline of *GPX4* and *GSS* transcripts in the combined group gives the possibility of inadequate scavenging activity of GSH and GPX4 detoxification mechanism against the huge ROS and lipid peroxide burden in the double group.

Ferroptosis induced by PRO treatment in HT-29 cells is coupled with activation of iron and lipid metabolism (canonical ferroptosis). Biochemically, the accumulation of intracellular and Mito ROS, reduction in MMP, and high MDA levels are features of ferroptosis upregulation in PRO-treated cells [44,45]. Consistent with these results, recent studies have reported that PRO increases intracellular ROS levels in human ovarian cancer cells [37], increases Mito ROS levels, and decreases MMP in isolated mitochondria (in vitro) [77]. Although the current study reported the ferroptotic role of PRO against HT-29 cancer cells for the first time, it was proven that PRO is an anti-ferroptosis agent that suppresses organ injury using an in vivo model by scavenging the lipid peroxyl radical [78]. Previous findings have suggested that PRO plays a selective role in the regulation of ferroptosis, which may be dependent on different pathological and tissue types.

Transcriptomic data showed that HT-29 cells treated with CAP underwent ferroptosis through induction of iron metabolism and system Xc^−^/GSH synthesis/GPX4 (canonical ferroptosis). Although CAP treatment caused ferroptosis in HT-29 cells, there were no detectable biochemical features of ferroptosis, such as elevated ROS, decreased MMP, or high MDA levels, in comparison to the control. This may be due to the absence of ferroptosis-dependent lipid peroxidation metabolism in the CAP-treated cells. This revealed that PRO, a nonselective β-blocker, might be a more efficient agent for inducing ferroptosis than CAP, a chemotherapeutic agent, in HT-29 cells.

In the literature, there is no reported evidence regarding the upregulation of ferroptosis cell death by these treatments (PRO and/or CAP) in in vivo and in vitro models of all cancer types, which reveals the novelty of our study.

Remarkably, we noted that dual treatment of HT-29 cells not only promoted ferroptosis but also stimulated the immune response. Moreover, ferroptosis has been found to cause immunogenic cell death [44,79]. Transcriptomic analysis of the co-treatment group revealed a significant downregulation of gene and protein expression by JAK-STAT signaling after IL-12 stimulation by decreasing the transcript level of the BOLA2B/LOC107984053 gene. The JAK-STAT signaling pathway participates in several cellular processes, including cell proliferation, apoptosis, cell survival, angiogenesis, invasion, migration, and the immune response [80,81]. A recent study reported that activation of the JAK/STAT signaling pathway leads to the development of resistance to BRAF inhibitors in BRAF^V600E^ thyroid carcinoma, which makes the JAK/STAT pathway a potential target for antitumor activity and to overcome drug resistance [82]. Moreover, intracellular transduction of JAK-STAT is activated by several cytokines, such as interleukins (IL_s_) and interferons (IFN) [81,83]. Notably, cytokines released by dead ferroptotic cells can induce both innate and adaptive immune responses [44,65]. JAK-STAT is one of the multiple inflammation-related signaling pathways that can lead to ferroptosis [84]. A recent study showed that activating the JAK/STAT pathway via IFN-γ leads to downregulation of genes related to ferroptosis induction in hepatocellular carcinoma (HCC) cell lines [85]. Another study reported that the use of certain double inhibitors can cause ferroptosis in cancer cells by inducing endogenous IFN-γ signaling via the STAT signaling pathway [86]. In our study, inhibition of the *BOLA2B/LOC107984053* transcript in PRO and combined groups of HT-29 cells may contribute to ferroptosis induction through the JAK/STAT pathway. *BOLA2B* is a newly mapped protein coding gene that plays an important role in iron regulation. The product of this molecule has been found to be overexpressed in tumors and can be utilized as a poor prognostic biomarker in several cancers [87]. Furthermore, the expression of BOLA2B is negatively correlated with immune cell infiltration in most cancers.

Based on our results, we suggest that the co-treatment group activated the immune response more than the PRO group, which might lead to ferroptosis-lipid peroxidation in cancer cells, which was verified by the increase in the related transcripts, ROS, and MDA levels. Moreover, evidence has shown that exposure to PRO, a nonselective β-blocker, activates the tumor microenvironment (in vivo) by increasing the intratumoral frequency of CD8+ T cells [88]. This study is consistent with our study on the activation of the immune response in tumors through PRO application.

In our study, the CRC model that showed a synergistic interaction between PRO and CAP was the HT-29 cell line with a mutation in the *TP53* gene (Supplementary Materials, Table S4). This mutation leads to impairment of the apoptotic cell death signaling pathway and the development of drug resistance in CRC [89]. In HT-29 cells, synergistic induction of ferroptotic cell death may occur through a compensatory mechanism that bypasses this genetic defect by targeting an alternative pathway. The potential molecular mechanism that may overcome *TP53* mutation-driven resistance and synergistically promote ferroptosis is by increasing total ROS, upregulating the transcripts of genes related to iron and lipid metabolism, elevating lipid peroxidation, and accelerating ferroptosis.

The anti-cancer effects of the double therapy were attributed to the induction of combined ferroptosis/necroptosis and immune response. In addition, the above results indicate that ferroptosis not only affects biochemical and metabolomic parameters but also crosslinks with other enriched signaling pathways, such as necroptosis and immune response.

## 5. Conclusions

Our findings demonstrated that the addition of combined treatment to HCT-116 and HT-29 cells produced differential effects on the cytotoxicity of these cell lines. Given that PRO is currently under clinical investigation [90], the synergism potential of combined treatment in HT-29 (*BRAF^V600E^*) may provide valuable insights for ongoing CRC research, especially for CRC cases with *BRAF^V600E^*. The mutations in the *BRAF* gene account for 8–12% of metastatic CRC (mt CRC) cases, and the variant *BRAF^V600E^* gene mutation represents the most common alteration to the *BRAF* gene with an aggressive phenotype. This mutation is associated with poor prognosis and a low response to therapy [91,92]. The application of PRO plus CAP therapy is consistent with current international guidelines for treating cases of BRAFV^600E^-mutant mt CRC. They recommend applying either a double or triple combination chemotherapy regimen with or without a certain inhibitor as the first-line treatment [92].

Our study is the first to report the promotion of ferroptosis using a combination therapy of PRO and CAP in HT-29 cells. Additionally, global metabolome analysis and RNA sequencing significantly enhanced our understanding of molecular targets, revealing novel potential targets for monotherapy and combination therapy in HT-29 cells. This research emphasizes the importance of precision medicine in cancer treatment. Moreover, evaluating the effects of PRO plus CAP in preclinical CRC models with different mutation profiles of proto-oncogenes and tumor suppressor genes could yield additional impacts on their therapeutic potential. Furthermore, the application of the PRO + CAP combination to other cancer types may help determine whether similar effects can be observed. Further research using multi-omics approaches, such as genomics and proteomics, is necessary to deepen our understanding of these mechanisms and expand the therapeutic potential of these treatments.

## Figures and Tables

**Table 1:** The IC_50_ values of PRO and CAP treatment for HCT-116 and HT-29 cell lines at 48 h.

Cell Line	PRO (µM)	CAP (mM)
HCT-116	129.26 ± 5.45	4.78 ± 0.07
HT-29	75.71 ± 12.58	1.94 ± 0.77

Data are expressed as the mean ± standard error of three independent experiments (n = 3).

**Table 2:** The combination index (CI) values of the dual therapy (PRO and CAP) for HCT-116 and HT-29 cell lines. The median effective doses (ED) were obtained from CompuSyn software and are expressed as (ED50, 75, 90, and 95). The type of drug interaction is determined based on CI theorem as follows: additive effect (CI = 1), synergism effect (CI < 1), and antagonism effect (CI > 1).

Cell Line	CI (ED50)	CI (ED75)	CI (ED90)	CI (ED95)	Type of Interaction
HCT-116	1.06 ± 0.06	1.04 ± 0.04	1.01 ± 0.01	0.94 ± 0.04	Additive
HT-29	0.85 ± 0.005	0.63 ± 0.04	0.47 ± 0.05	0.33 ± 0.002	Highly synergism

**Table 3:** Ferroptosis pathway analysis of DEGs) in HT-29 adenocarcinoma cells treated with PRO and/or CAP.

Treatment	Gene Symbol	LFC	Expression Level Based on RNA-Seq	*p*-Value
PRO	*ACSL1*	0.88	Up	0.01
	*FTH1P2*	0.99	Up	0.04
CAP	*FTH1P2*	1.15	Up	0.03
	*FTH1P23*	1.12	Up	0.02
	*FTL*	1.03	Up	0.02
	*GCLM*	0.78	Up	0.03
	*HMOX1*	1.93	Up	0.001
	*SLC3A2*	0.70	Up	0.04
PRO + CAP	*ACSL1*	0.94	Up	0.008
	*FTH1*	0.86	Up	0.04
	*FTH1P2*	1.18	Up	0.01
	*FTH1P8*	1.10	Up	0.02
	*FTH1P23*	1.00	Up	0.04
	*GCLM*	0.99	Up	0.007
	*HMOX1*	1.39	Up	0.005
	*SLC7A11*	0.80	Up	0.03

**Table 4:** The enriched metabolic pathways (mitochondrial electron transport chain and phosphatidylcholine biosynthesis) for HT29 cell line after the treatments with PRO plus CAP.

Pathway Name	Total	Hits	*p*-Value	Relative Metabolites
Mitochondrial electron transport chain	19	5	1.71 × 10^−3^	DHAPH, GA3P, glyceric acid-1,3-bisphosphate, fumarate, and FAD.
Phosphatidylcholine biosynthesis	14	5	6.65 × 10^−3^	Cytosine triphosphate (CTP), cytosine monophosphate (CMP), SAM, and phosphocholines (PCs).

## Data Availability

The data presented in this study are available upon request from the corresponding author.

## Supplementary Materials

The following supporting information can be downloaded at https://www.mdpi.com/article/10.3390/cancers17091470/s1, Table S1. The top 50 up/downregulated differentially expressed genes (DEGs) based on RNA-sequencing data analysis of HT-29 after the treatment with PRO and/or CAP. Table S2. Pathway enrichment analysis of differentially expressed genes (DEGs) in HT-29 cells treated with PRO + CAP. Table S3. Pathway enrichment analysis of differentially expressed genes (DEGs) in HT-29 cells treated with PRO. Table S4. Origin and molecular features of the colorectal cell lines (HCT-116 and HT-29). Figure S1. The one-way ANOVA and post-hoc analysis results calculated by MetaboAnalyst 5.0 for HT-29 cell line.
