# Correction: Alzahrani et al. Propranolol and Capecitabine Synergy on Inducing Ferroptosis in Human Colorectal Cancer Cells: Potential Implications in Cancer Therapy. *Cancers* 2025, *17*, 1470

**DOI:** 10.3390/cancers17213557

**Published:** 2025-11-03

**Authors:** Shiekhah Mohammad Alzahrani, Huda Abdulaziz Al Doghaither, Hind Ali Alkhatabi, Mohammad Abdullah Basabrain, Peter Natesan Pushparaj

**Affiliations:** 1 Biochemistry Department, Faculty of Science, King Abdulaziz University, Jeddah 21589 P.O. Box 80200, Saudi Arabia; salzahrani1329@stu.kau.edu.sa; 2 Institute of Genomic Medicine Sciences, Faculty of Applied Medical Sciences, King Abdulaziz University, Jeddah P.O. Box 21589, Saudi Arabia; mohammad.basabrain@gmail.com (M.A.B.); pnatesan@kau.edu.sa (P.N.P.); 3 Department of Biological Science, College of Science, University of Jeddah, Jeddah P.O. Box 21589, Saudi Arabia; haalkhatabi@uj.edu.sa; 4 Department of Medical Laboratory Technology, Faculty of Applied Medical Sciences, King Abdulaziz University, Jeddah P.O. Box 21589, Saudi Arabia

## 1. Affiliations

In the original publication [1], there was an error with the affiliation (1). The P.O. box number has been added. 

## 2. Figure/Table Legend

In the original publication [1], there was a mistake with Figures 3 and 6. **For Figure 3, the yellow highlight boxes have been removed and for Figure 6, GCLC has been replaced with GCLM and SLC3A2 with SLC7A11**. The figures appear below.

**Figure 3:**
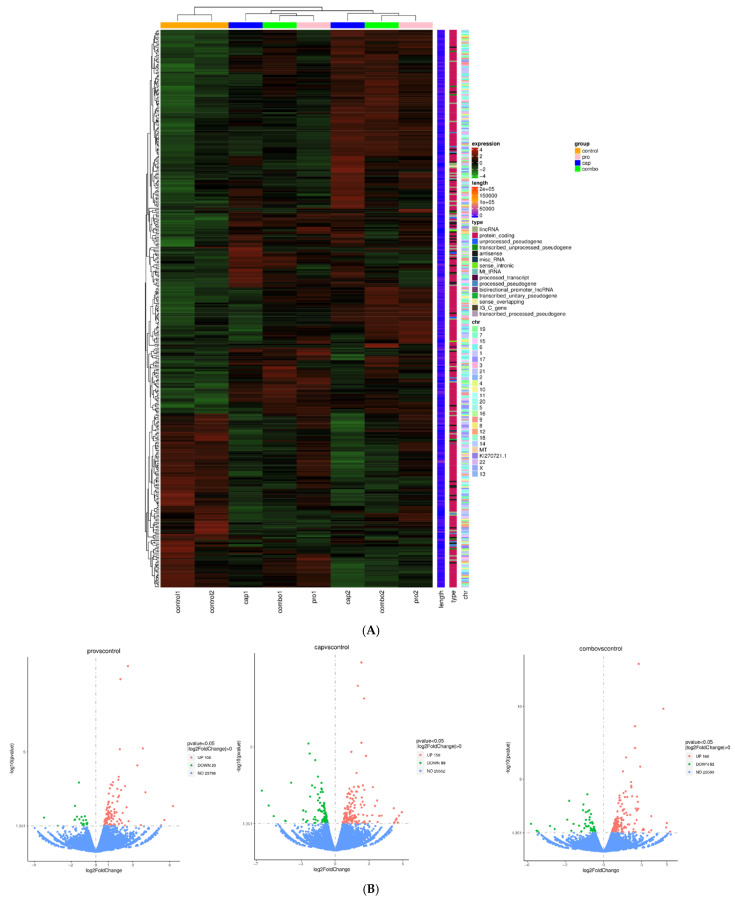

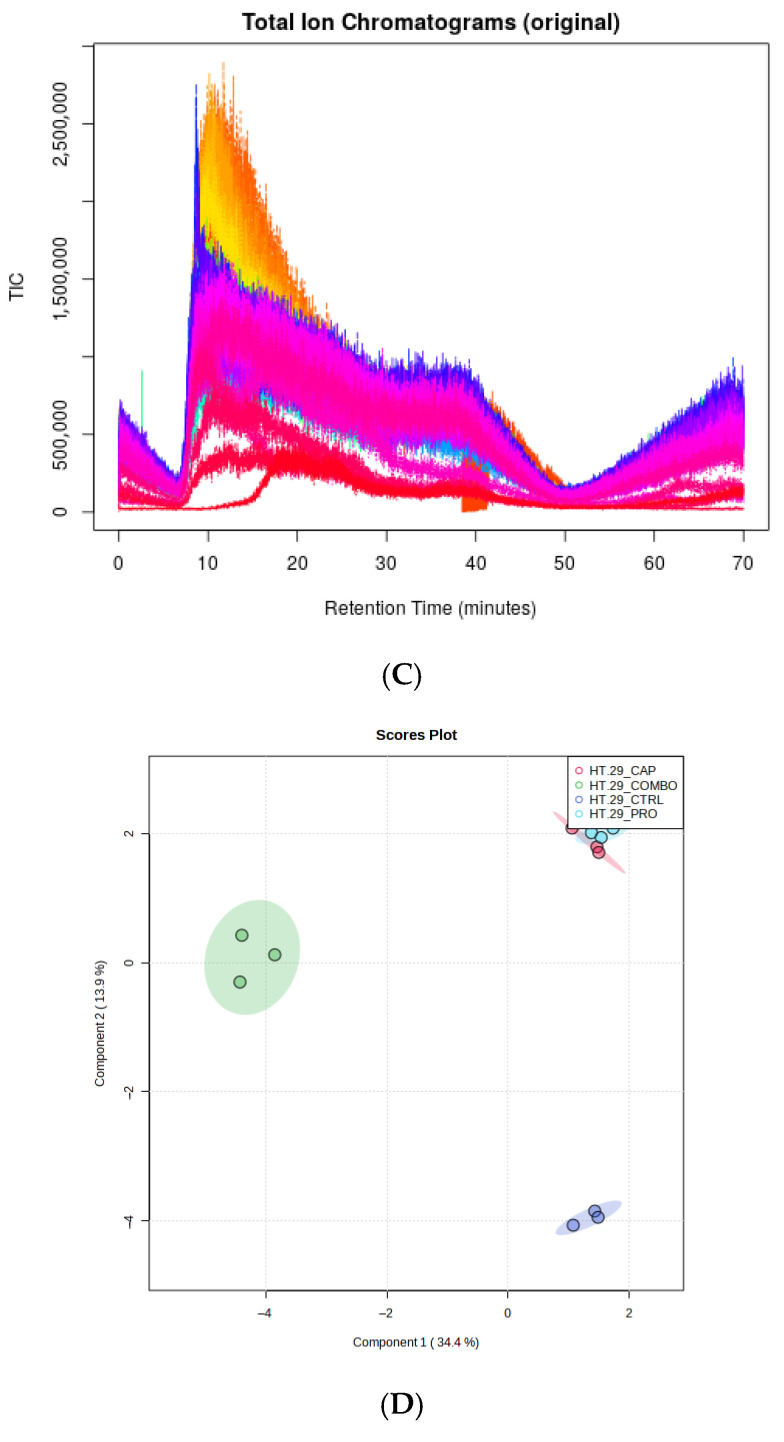

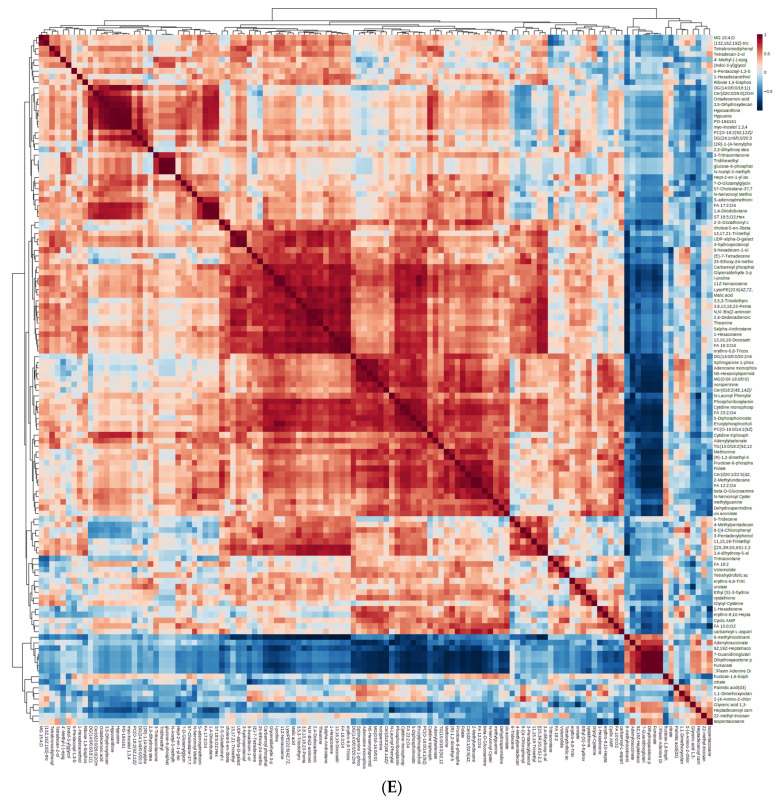

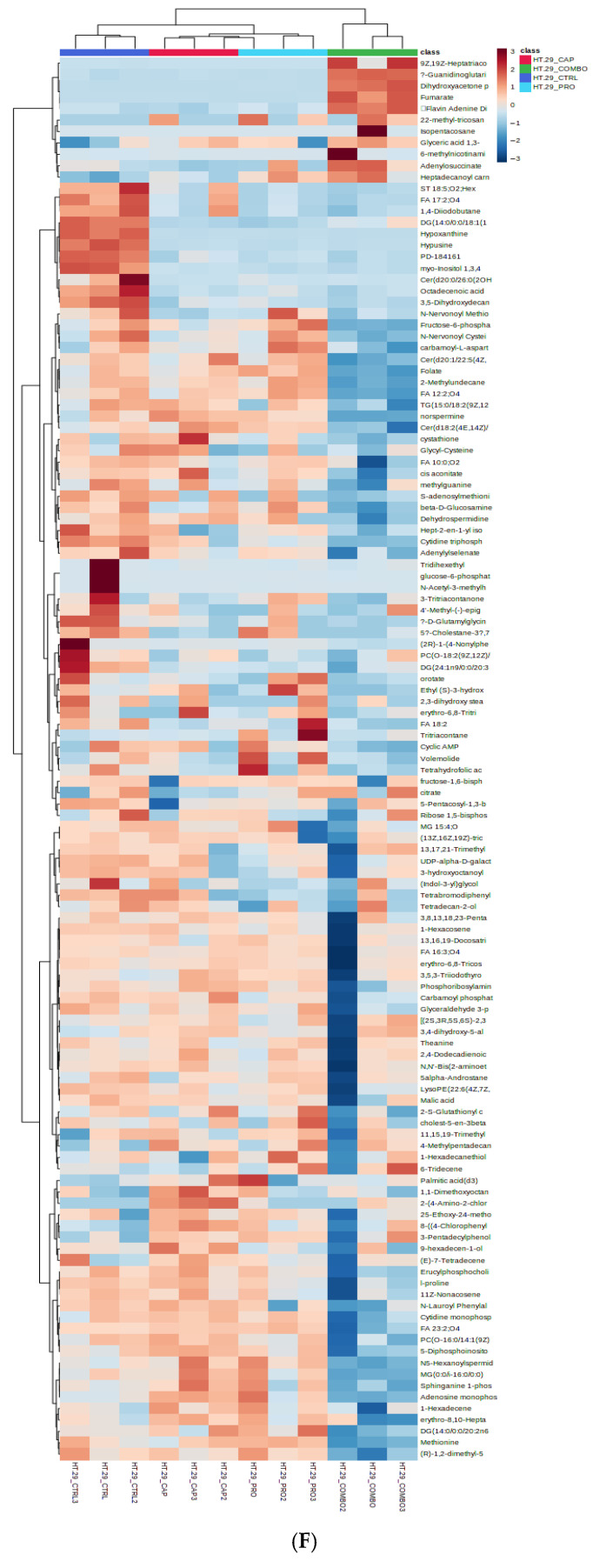

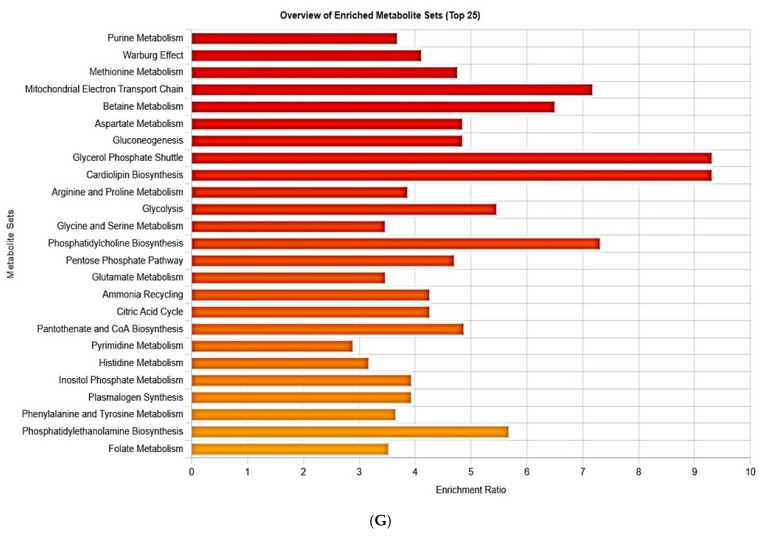
Overall view of the transcriptomic and metabolomic profiles of HT-29 cells after treatment with PRO and/or CAP compared with untreated cells. (**A**) Heatmap showing the expression pattern of genes in HT-29-treated groups versus the control group. (**B**) Volcano plot showing the positions of differentially upregulated and downregulated genes in the PRO, CAP, and PRO + CAP groups versus the control group. (**C**) Total ion chromatograms of the extracted metabolites from treated and untreated HT-29 cells, which were run in LTQ-XL linear ion trap LC-MS. (**D**) Principal component analysis (PCA) of comprehensive metabolites from treated and untreated HT-29 cells. (**E**) Correlation heatmaps of treated and untreated HT-29 cells. (**F**) Heatmaps of differentially expressed metabolites in treated and untreated HT-29 cells. (**G**) The top twenty-five pathways enriched in the metabolome analysis of treated and untreated HT-29 cell lines.

**Figure 6:**
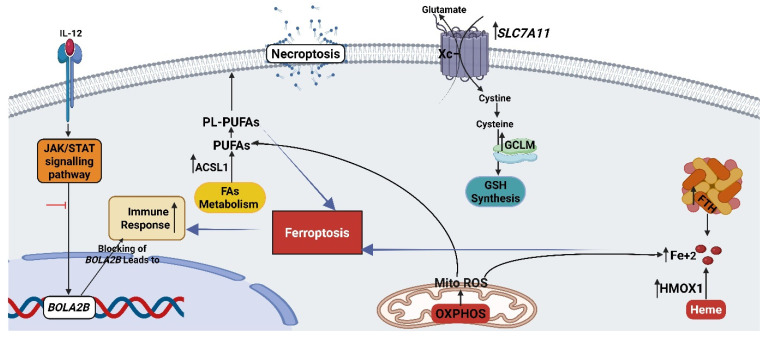
Proposed mechanism of combined therapy in HT-29 cell line based on RNA-seq results. The figure was created with BioRender (www.biorender.com).

## 3. Supplementary Files

In the original supplementary files, there was a typo for the reference list used in Table S4. The name of the reference list has been changed from “References of Table S6” to “References of Table S4”. 

The scientific conclusions are unaffected. This correction was approved by the Academic Editor. The original publication has also been updated.
