# Supplementary material for: Propranolol and Capecitabine Synergy on Inducing Ferroptosis in Human Colorectal Cancer Cells: Potential Implications in Cancer Therapy
Source: Cancers (Basel). 2025 Apr 27;17(9):1470. doi: 10.3390/cancers17091470 (PMC12071015; doi:10.3390/cancers17091470)
Supplement: Supplementary file 1 [file cancers-17-01470-s001.zip › cancers-3518284-supplementary.pdf]

Supplementary Tables

Table S1. The top 50 up/downregulated differentially expressed gene (DEGs) based on RNA-sequencing data analysis of HT-29 after the treatment with PRO and/or CAP.

A) PRO

gene_id	log2FoldChange	pvalue	padj	gene_name	gene_chr
ENSG00000268621	2.610419932	5.12E-10	1.33E-05	IGFL2-AS1	19
ENSG00000198074	2.002837517	2.29E-09	2.97E-05	AKR1B10	7
ENSG00000166831	3.81719113	6.69E-06	0.047925965	RBPMS2	15
ENSG00000181577	1.945102708	7.40E-06	0.047925965	C6orf223	6
ENSG00000268879	3.373841047	4.71E-05	0.243964773	IGFL1P1	19
ENSG00000171903	1.862893701	0.000176571	0.762844562	CYP4F11	19
ENSG00000143839	1.835846287	0.00024697	0.914563759	REN	1
ENSG00000169174	1.391204079	0.000325503	0.984198607	PCSK9	1
ENSG00000277053	-1.370332569	0.000341709	0.984198607	GTF2IP1	7
ENSG00000169710	1.223508381	0.00042491	0.99998842	FASN	17
ENSG00000124762	1.349026065	0.000465013	0.99998842	CDKN1A	6
ENSG00000171658	2.37053093	0.000514702	0.99998842	NMRAL2P	3
ENSG00000160179	1.139751281	0.000662915	0.99998842	ABCG1	21
ENSG00000115295	1.744498077	0.000696777	0.99998842	CLIP4	2
ENSG00000118785	2.057144364	0.00080689	0.99998842	SPP1	4
ENSG00000267922	4.031260729	0.001047746	0.99998842	AC007785.1	19
ENSG00000099194	1.04986676	0.001210392	0.99998842	SCD	10
ENSG00000141574	1.891041507	0.001553528	0.99998842	SECTM1	17
ENSG00000260196	1.40714863	0.001640704	0.99998842	AC124798.1	11
ENSG00000131069	1.813207031	0.00185505	0.99998842	ACSS2	20
ENSG00000164867	1.744471039	0.002972117	0.99998842	NOS3	7
ENSG00000112972	1.041160204	0.003070322	0.99998842	HMGCS1	5
ENSG00000138166	1.031088135	0.003410454	0.99998842	DUSP5	10
ENSG00000184371	1.339820267	0.003550695	0.99998842	CSF1	1
ENSG00000160285	0.984630369	0.004249071	0.99998842	LSS	21
ENSG00000134240	-1.695191458	0.004970453	0.99998842	HMGCS2	1
ENSG00000125571	6.270907246	0.00510908	0.99998842	IL37	2
ENSG00000090339	1.136957372	0.005532444	0.99998842	ICAM1	19
ENSG00000130477	2.494675946	0.005909535	0.99998842	UNC13A	19
ENSG00000101384	1.083873592	0.006045637	0.99998842	JAG1	20
ENSG00000005001	0.926420449	0.006057972	0.99998842	PRSS22	16
ENSG00000225630	0.920061227	0.006396653	0.99998842	MTND2P28	1
ENSG00000135842	1.499286051	0.007367833	0.99998842	FAM129A	1
ENSG00000161011	0.859864116	0.007880734	0.99998842	SQSTM1	5
ENSG00000134324	1.0019794	0.009072781	0.99998842	LPIN1	2
ENSG00000165029	1.239931133	0.009201647	0.99998842	ABCA1	9
ENSG00000023839	1.517975677	0.010085413	0.99998842	ABCC2	10
ENSG00000176945	1.065983459	0.010440397	0.99998842	MUC20	3
ENSG00000164949	1.69607802	0.010502929	0.99998842	GEM	8
ENSG00000186480	0.988642124	0.010802545	0.99998842	INSIG1	7
ENSG00000144130	2.184913055	0.011344652	0.99998842	NT5DC4	2
ENSG00000137440	1.521907314	0.011545154	0.99998842	FGFBP1	4
ENSG00000135919	1.133484131	0.011721546	0.99998842	SERPINE2	2
ENSG00000186806	1.021473723	0.012445671	0.99998842	VSIG10L	19
ENSG00000151726	0.8767969	0.01244646	0.99998842	ACSL1	4
ENSG00000188211	0.866885969	0.013219475	0.99998842	NCR3LG1	11
ENSG00000140853	1.254544282	0.013374727	0.99998842	NLRC5	16
ENSG00000181652	1.306878334	0.013729053	0.99998842	ATG9B	7
ENSG00000137193	0.99124029	0.013932681	0.99998842	PIM1	6
ENSG00000102575	1.287491227	0.015291968	0.99998842	ACP5	19

B) CAP

gene_id	log2FoldChange	pvalue	padj	gene_name	gene_chr
ENSG00000275896	2.333667204	8.73E-10	2.25E-05	PRSS2	7
ENSG00000198074	2.006921059	1.16E-08	0.000149857	AKR1B10	7
ENSG00000204983	2.575851796	4.74E-08	0.000407528	PRSS1	7
ENSG00000135842	2.332464112	6.41E-06	0.03603448	FAM129A	1
ENSG00000161798	-2.372503199	6.98E-06	0.03603448	AQP5	12
ENSG00000160179	1.436471541	1.76E-05	0.075737932	ABCG1	21
ENSG00000257588	-2.258123062	2.12E-05	0.078131455	AC025154.2	12
ENSG00000171658	2.731870564	2.75E-05	0.088791625	NMRAL2P	3
ENSG00000198829	-1.771352112	4.39E-05	0.125961319	SUCNR1	3
ENSG00000197405	1.900675648	0.000182194	0.4702233	C5AR1	19
ENSG00000196950	-1.527133498	0.000275312	0.645956496	SLC39A10	2
ENSG00000188153	-3.906376165	0.000541031	0.999919565	COL4A5	X
ENSG00000068489	-1.214122103	0.000547899	0.999919565	PRR11	17
ENSG00000204740	-2.053666531	0.000729799	0.999919565	MALRD1	10
ENSG00000186529	2.45747281	0.000730073	0.999919565	CYP4F3	19
ENSG00000118785	2.035045527	0.000815743	0.999919565	SPP1	4
ENSG00000236039	2.095218747	0.000862924	0.999919565	AC019117.1	7
ENSG00000078018	1.66428742	0.000865971	0.999919565	MAP2	2
ENSG00000166831	3.347679371	0.000880611	0.999919565	RBPMS2	15
ENSG00000135472	-1.798782129	0.00092279	0.999919565	FAIM2	12
ENSG00000072571	-1.221788484	0.001014904	0.999919565	HMMR	5
ENSG00000113369	1.263297813	0.001041302	0.999919565	ARRDC3	5
ENSG00000140451	-1.185656973	0.001166613	0.999919565	PIF1	15
ENSG00000073737	2.587558503	0.00118317	0.999919565	DHRS9	2
ENSG00000224659	-6.512258348	0.001300955	0.999919565	GAGE12J	X
ENSG00000125775	1.424571318	0.001354706	0.999919565	SDCBP2	20
ENSG00000186204	1.862855275	0.001416636	0.999919565	CYP4F12	19
ENSG00000100292	1.930737171	0.001459763	0.999919565	HMOX1	22
ENSG00000165029	1.493842015	0.001461777	0.999919565	ABCA1	9
ENSG00000179348	-1.090170664	0.001506747	0.999919565	GATA2	3
ENSG00000094755	-1.28464017	0.001522494	0.999919565	GABRP	5
ENSG00000124762	1.21500708	0.001704675	0.999919565	CDKN1A	6
ENSG00000137801	1.109225417	0.001869056	0.999919565	THBS1	15
ENSG00000125148	1.321350316	0.001923449	0.999919565	MT2A	16
ENSG00000176244	-1.532189844	0.001941352	0.999919565	ACBD7	10
ENSG00000248323	1.656355506	0.002196371	0.999919565	LUCAT1	5
ENSG00000167580	-2.217339715	0.002526167	0.999919565	AQP2	12
ENSG00000171431	1.050471286	0.002601418	0.999919565	KRT20	17
ENSG00000161011	1.098908127	0.002657474	0.999919565	SQSTM1	5
ENSG00000249839	2.363381753	0.002757505	0.999919565	AC011330.1	15
ENSG00000078401	-1.389805366	0.002872226	0.999919565	EDN1	6
ENSG00000125144	1.830507807	0.00287399	0.999919565	MT1G	16
ENSG00000198768	1.357389765	0.003026535	0.999919565	APCDD1L	20
ENSG00000185567	1.550012779	0.003194799	0.999919565	AHNAK2	14
ENSG00000224259	1.542455542	0.003385658	0.999919565	LINC01133	1
ENSG00000176912	-1.515457509	0.003853059	0.999919565	TYMSOS	18
ENSG00000163053	3.729897292	0.003889906	0.999919565	SLC16A14	2
ENSG00000140961	1.664068119	0.004162484	0.999919565	OSGIN1	16
ENSG00000130477	2.434861157	0.004432355	0.999919565	UNC13A	19
ENSG00000265415	-1.338295429	0.004990621	0.999919565	AC099850.3	17

C) PRO + CAP

gene_id	log2FoldChange	pvalue	padj	gene_name	gene_chr
ENSG00000198074	2.777269408	1.16E-13	1.77E-09	AKR1B10	7
ENSG00000166831	4.721692149	1.44E-10	1.10E-06	RBPMS2	15
ENSG00000268621	2.483197286	2.29E-09	1.17E-05	IGFL2-AS1	19
ENSG00000171903	2.484366126	7.28E-08	0.000278659	CYP4F11	19
ENSG00000124762	1.777781096	3.22E-07	0.000986092	CDKN1A	6
ENSG00000171658	2.894471504	1.39E-06	0.003547905	NMRAL2P	3
ENSG00000160179	1.527089207	2.94E-06	0.006435817	ABCG1	21
ENSG00000248323	2.170416938	1.60E-05	0.030551558	LUCAT1	5
ENSG00000135842	2.183582678	2.84E-05	0.048274894	FAM129A	1
ENSG00000143839	2.191842871	3.82E-05	0.058504058	REN	1
ENSG00000198768	1.74228552	7.04E-05	0.098004514	APCDD1L	20
ENSG00000125775	1.57228377	9.14E-05	0.116500402	SDCBP2	20
ENSG00000198829	-1.283652298	0.00011297	0.132983534	SUCNR1	3
ENSG00000161011	1.25802005	0.000136103	0.148592869	SQSTM1	5
ENSG00000118785	2.254882188	0.000152278	0.148592869	SPP1	4
ENSG00000186529	2.637271214	0.000161418	0.148592869	CYP4F3	19
ENSG00000165029	1.679712218	0.000172906	0.148592869	ABCA1	9
ENSG00000185567	1.861532148	0.000174781	0.148592869	AHNAK2	14
ENSG00000169710	1.252278012	0.000196653	0.158388578	FASN	17
ENSG00000231290	1.327286876	0.000286668	0.216172932	APCDD1L-DT	20
ENSG00000171431	1.255378463	0.00029665	0.216172932	KRT20	17
ENSG00000205572	-2.720466125	0.000317332	0.220733331	SERF1B	5
ENSG00000073737	2.709276765	0.000371889	0.247435696	DHRS9	2
ENSG00000099194	1.169198375	0.000463066	0.295262273	SCD	10
ENSG00000160712	1.455499896	0.000527353	0.322803215	IL6R	1
ENSG00000135472	-1.59279813	0.000563067	0.331408487	FAIM2	12
ENSG00000101384	1.337697291	0.000775051	0.439281625	JAG1	20
ENSG00000144130	2.541398623	0.001070074	0.578834215	NT5DC4	2
ENSG00000235899	2.732006815	0.001096922	0.578834215	LINC01564	6
ENSG00000115295	1.615780677	0.001208206	0.601054507	CLIP4	2
ENSG00000134278	1.563954868	0.001241667	0.601054507	SPIRE1	18
ENSG00000121895	2.688451236	0.001256861	0.601054507	TMEM156	4
ENSG00000140451	-1.128908312	0.001387129	0.623177033	PIF1	15
ENSG00000257588	-1.632760132	0.001410353	0.623177033	AC025154.2	12
ENSG00000204983	1.490700652	0.001480966	0.623177033	PRSS1	7
ENSG00000181577	1.521036327	0.001491293	0.623177033	C6orf223	6
ENSG00000130477	2.617310006	0.001506734	0.623177033	UNC13A	19
ENSG00000138166	1.093716707	0.001676861	0.666350843	DUSP5	10
ENSG00000277053	-1.238995081	0.001701224	0.666350843	GTF2IP1	7
ENSG00000001084	1.092836736	0.001741752	0.666350843	GCLC	6
ENSG00000078401	-1.400424262	0.00189983	0.7091	EDN1	6
ENSG00000164949	1.783058647	0.002096949	0.764038442	GEM	8
ENSG00000140961	1.627818215	0.00245839	0.874901121	OSGIN1	16
ENSG00000179348	-1.000730914	0.002574979	0.895565937	GATA2	3
ENSG00000231683	2.100812026	0.002933883	0.997715724	AL033397.1	6
ENSG00000236039	1.868298636	0.00307458	0.999869802	AC019117.1	7
ENSG00000241860	3.766749026	0.003621104	0.999869802	AL627309.5	1
ENSG00000113369	1.094401437	0.004084142	0.999869802	ARRDC3	5
ENSG00000184110	-1.102774134	0.004300881	0.999869802	EIF3C	16
ENSG00000078018	1.422336457	0.004561381	0.999869802	MAP2	2

Table S2. Pathway enrichment analysis of differentially expressed genes (DEGs) in HT-29 cells treated with PRO+CAP.

Term	Source	Term- ID	Related genes	LFC	Expression Level based on RNA-seq	*P*-value
**Necroptosis**	KEGG-Up	hsa04217	*SQSTM1, FTH1P2, FTH1P8, FTH1P23,* and *FTH1*	#	Up	0.04
**Gene and protein expression by JAK-STAT signaling after Interleukin-12 stimulation**	Reactome-Down	R-HSA-8950505	*BOLA2B/LOC107984053*	-3.86	Down	0.004

Table S3. Pathway enrichment analysis of differentially expressed genes (DEGs) in HT-29 cells treated with PRO.

Term	Source	Term- ID	Related genes	LFC	Expression Level based on RNA-seq	*P*-value
**Gene and protein expression by JAK-STAT signaling after interleukin-12 stimulation**	Reactome-Down	R-HSA-8950505	*BOLA2B/ LOC107984053*	-4.2	Down	0.01

Table S4. Origin and molecular features of the colorectal cell lines (HCT-116 and HT-29).

Term	HCT-116	HT-29	References
**Disease**	Colorectal carcinoma	Colorectal adenocarcinoma	[1-3]
**Metastasis/Stage**	Primary tumor/Duke stage D	A primary tumor/Dukes' C	[1-4]
**Doubling time of growth**	Approximately 18 h	24 h	[5-8]
**Microsatellite (MS) stability status**	MSI-high	MSS	[4, 9]
**CIMP Panel 1/2**	+/+	+/+	[4]
**Chromosomal instability (CIN)**	*CIN*−	*CIN*+	[4]
***KRAS* mutation status**	G13D	wt	[4]
***BRAF* mutation status**	wt	V600E	[4]
***PIK3CA* mutation status**	H1047R	P449T	[4]
***PTEN* mutation status**	wt	wt	[4]
***TP53* mutation status**	wt	R273H	[4]
**5-FU response**	5-FU resistant	5-FU sensitive	[10-12]
**Cell morphology**	Homogenous cell line with epithelial morphology	Heterogenous cell line with a multilayer of unpolarized cells	[13, 14]

References of Table S4:

1. Fogh, J., Trempe, G., 1975. New human tumor cell lines. In *Human tumor cells in vitro* (pp. 115-159). Boston, MA: Springer US. DOI: https://doi.org/10.1007/978-1-4757-1647-4_5
2. Leibovitz, A., Stinson, J.C., McCombs III, W.B., McCoy, C.E., Mazur, K.C., Mabry, N.D., 1976. Classification of human colorectal adenocarcinoma cell lines. Cancer research. 36 (12), 4562-4569. PMID: 1000501
3. Brattain, M.G., Brattain, D.E., Fine, W.D., Khaled, F.M., Marks, M.E., Kimball, P.M., Arcolano, L.A., Danbury, B.H., 1981. Initiation and characterization of cultures of human colonic carcinoma with different biological characteristics utilizing feeder layers of confluent fibroblasts. Oncodevelopmental biology and medicine: the journal of the International Society for Oncodevelopmental Biology and Medicine. 2 (5), 355-366. PMID: 7329817
4. Ahmed, D., Eide, P.W., Eilertsen, I.A., Danielsen, S.A., Eknæs, M., Hektoen, M., Lind, G.E., Lothe, R., 2013. Epigenetic and genetic features of 24 colon cancer cell lines. Oncogenesis. 2 (9), e71. DOI: https://doi.org/10.1038/oncsis.2013.35
5. Forgue-Lafitte, M.E., Coudray, A.M., Bréant, B., Mešter, J., 1989. Proliferation of the human colon carcinoma cell line HT29: autocrine growth and deregulated expression of the c-myc oncogene. Cancer research. 49 (23), 6566-6571. PMID: 2684395
6. Cowley, G.S., Weir, B.A., Vazquez, F., Tamayo, P., Scott, J.A., Rusin, S., East-Seletsky, A., Ali, L.D., Gerath, W.F., Pantel, S.E., Lizotte, P.H., 2014. Parallel genome-scale loss of function screens in 216 cancer cell lines for the identification of context-specific genetic dependencies. Scientific data. 1 (1), 1-12. DOI: https://doi.org/10.1038/sdata.2014.35
7. Schulte am Esch, J., Windmöller, B.A., Hanewinkel, J., Storm, J., Förster, C., Wilkens, L., Krüger, M., Kaltschmidt, B., Kaltschmidt, C., 2020. Isolation and characterization of two novel colorectal cancer cell lines, containing a subpopulation with potential stem-like properties: Treatment options by MYC/NMYC inhibition. Cancers. 12 (9), 2582. DOI: https://doi.org/10.3390/cancers12092582
8. Home (no date) SIB Swiss Institute of Bioinformatics. Available online at: https://web.expasy.org/cellosaurus/CVCL_0547 (Accessed: March 14, 2023) https://web.expasy.org/cellosaurus/CVCL_0547

9. Cottu, P.H., Muzeau, F., Estreicher, A., Fléjou, J.F., Iggo, R., Thomas, G., Hamelin, R., 1996. Inverse correlation between RER+ status and p53 mutation in colorectal cancer cell lines. Oncogene. 13 (12), 2727-2730. PMID: 9000147
10. Kunnumakkara, A.B., Diagaradjane, P., Anand, P., Kuzhuvelil, H.B., Deorukhkar, A., Gelovani, J., Guha, S., Krishnan, S., Aggarwal, B.B., 2009. Curcumin sensitizes human colorectal cancer to capecitabine by modulation of cyclin D1, COX-2, MMP-9, VEGF and CXCR4 expression in an orthotopic mouse model. Int. J. Cancer Res. 125 (9), 2187-2197. DOI: https://doi.org/10.1002/ijc.24593
11. Mhaidat, N.M., Bouklihacene, M., Thorne, R.F., 2014. 5-Fluorouracil-induced apoptosis in colorectal cancer cells is caspase-9-dependent and mediated by activation of protein kinase C-δ. Oncol. lett. 8 (2), 699-704. DOI: https://doi.org/10.3892/ol.2014.2211
12. Chin, C.C., Li, J.M., Lee, K.F., Huang, Y.C., Wang, K.C., Lai, H.C., Cheng, C.C., Kuo, Y.H., Shi, C.S., 2016. Selective β2-AR blockage suppresses colorectal cancer growth through regulation of EGFR-Akt/ERK1/2 signaling, G1-phase arrest, and apoptosis. J. Cell Physiol. 231 (2), 459-472. DOI: https://doi.org/10.1002/jcp.25092
13. HCT 116 cell line (no date) Creative Biogene. Available online at: https://www.creative-biogene.com/support/hct-116-cell-line.html (Accessed: March 14, 2023) https://www.creative-biogene.com/support/hct-116-cell-line.html
14. Biazik, J.M., Jahn, K.A., Su, Y., Wu, Y.N., Braet, F., 2010. Unlocking the ultrastructure of colorectal cancer cells in vitro using selective staining. World J. Gastroenterol. 16 (22), 2743. DOI: 10.3748/wjg.v16.i22.2743

Supplementary Figures

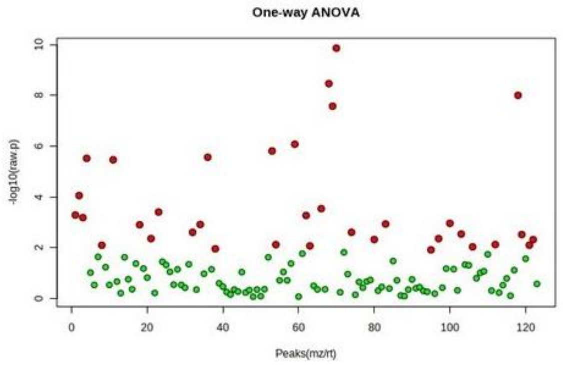

Fig. S1. The one-way ANOVA and post-hoc analysis results calculated by MetaboAnalyst 5.0 for HT-29 cell line.
